# From Longevity Genetics to Precision Interventions: Integrating Nutrigenomics and Epigenetic Mechanisms of Ageing

**DOI:** 10.3390/genes17060681

**Published:** 2026-06-10

**Authors:** Lorin-Manuel Pîrlog, Andreea Cătană, Adela-Diana Pitforodeschi, Alissia Nicoleta Pilatec, Rareș-Mihai Băilă, Irina Rusu, Mariela-Sanda Militaru, Irina Ioana Iordănescu, Andrada-Adelaida Belbe

**Affiliations:** 1 Department of Molecular Sciences, Faculty of Medicine, “Iuliu Hațieganu” University of Medicine and Pharmacy, 400012 Cluj-Napoca, Romania; lorin.pirlog@gmail.com (L.-M.P.); adela.diana.p1@gmail.com (A.-D.P.); pilatecalissia@gmail.com (A.N.P.); raresbaila@gmail.com (R.-M.B.); irinarusu2003@gmail.com (I.R.); sanda.militaru@umfcluj.ro (M.-S.M.); adelaidapatrascanu@gmail.com (A.-A.B.); 2 Department of Medical Genetics, Clinical Emergency Hospital for Children, 400370 Cluj-Napoca, Romania; 3 Regional Laboratory Cluj-Napoca, Department of Medical Genetics, Regina Maria Health Private Network, 400363 Cluj-Napoca, Romania; 4 Department of Oncogenetics, “Prof. Dr. I. Chiricuță” Institute of Oncology, 400015 Cluj-Napoca, Romania; 5 Genetic Centre Laboratory, Department of Medical Genetics, Regina Maria Health Private Network, 011376 Bucharest, Romania; irina.iordanescu@reginamaria.ro

**Keywords:** longevity genetics, nutrigenomics, epigenetic ageing, precision geroscience, DNA methylation, epigenetic clocks, *FOXO3*, *APOE*, telomere biology, gene–environment interactions

## Abstract

Human ageing and longevity are increasingly understood as biologically integrated and heterogeneous processes shaped by interactions among genetic susceptibility, epigenetic remodelling, and environmental modulation. This narrative review examines these interconnections within a nutrigenomic framework, with particular emphasis on how inherited variation and epigenetic plasticity may influence responses to ageing-related interventions. A structured literature search was conducted in PubMed, Scopus, Web of Science, and Embase, focusing on English-language studies published during the last 10 years. The review was organized into three major domains: (i) genetic determinants of longevity, (ii) epigenetic mechanisms of ageing, and (iii) intervention-responsive pathways relevant to precision geroscience. Current evidence supports a polygenic model of longevity in which loci such as *FOXO3* and *APOE* show the most consistent human associations, while telomere maintenance, insulin/IGF-1 and mTOR signalling, sirtuins, Klotho, inflammatory mediators, and DNA repair remain biologically important but variably supported at the variant level. Epigenetic mechanisms, including DNA methylation drift, epigenetic clocks, histone modifications, chromatin remodelling, heterochromatin loss, and non-coding RNA regulation, provide an environmentally responsive interface linking genetic background to ageing phenotypes. Nutritional, pharmacological, behavioural, and circadian interventions converge on overlapping molecular pathways involving AMPK, mTOR, FOXO, sirtuins, autophagy, mitochondrial maintenance, and inflammatory signalling, although human evidence remains heterogeneous and biomarker modulation should not be equated with clinically meaningful slowing of organismal ageing. Overall, this review highlights the value of integrating genetics, epigenetics, and intervention biology to support a more cautious and translationally relevant model of healthy ageing. It also underscores the need for precision nutrigeroscience approaches that account for tissue context, baseline physiology, and inter-individual molecular variability.

## 1. Introduction

Population ageing has intensified interest in the biological mechanisms underlying lifespan and health span. Ageing is now viewed as a heterogeneous process influenced by genetic, epigenetic, environmental, metabolic, inflammatory, and stochastic factors. Although longevity is only modestly heritable, estimated at 12–25%, genetic variation still contributes to survival differences alongside strong lifestyle and environmental influences [1,2,3,4].

Current evidence supports a polygenic model of longevity, involving multiple pathways such as cardiometabolic regulation, neurodegeneration, immune function, stress resistance, nutrient sensing, telomere maintenance, DNA repair, and genome stability [1,2,3,5]. *FOXO3* and *APOE* remain among the most consistently replicated longevity-associated loci, while insulin/IGF-1 signalling, mTOR, sirtuins, Klotho, inflammatory mediators, telomere biology, and DNA repair pathways are also biologically important, despite variable evidence at the variant level [2,3,5,6,7,8,9,10,11,12,13,14,15,16,17,18,19,20,21,22,23,24,25,26,27,28,29,30,31,32,33,34,35,36,37,38,39,40,41,42,43,44,45,46,47,48,49,50,51,52,53,54,55,56,57,58,59,60,61,62,63,64,65,66,67,68,69,70,71,72,73,74,75,76,77,78,79,80,81,82,83,84,85,86].

Epigenetic mechanisms provide a major link between inherited predisposition and environmental influence. Ageing is associated with DNA methylation drift, altered histone modifications, chromatin remodelling, heterochromatin loss, non-coding RNA dysregulation, and transposable element derepression [17,18,19,20,21,22]. These processes contribute to transcriptional instability, impaired cellular identity, inflammation, and reduced stress resilience. Epigenetic changes are also responsive to diet and metabolic state, making DNA methylation clocks and related biomarkers central tools in ageing research [17,18,23,24,25,26,27,28,87,88,89,90,91,92,93,94,95,96,97,98,99,100,101,102,103,104,105,106,107,108,109,110,111,112,113,114,115,116,117,118,119,120,121,122,123,124,125,126,127,128,129,130,131,132,133,134,135,136,137,138,139,140,141,142,143,144,145,146,147,148,149,150,151,152,153,154,155,156,157,158,159,160,161,162,163,164,165,166,167,168,169,170,171,172,173,174,175,176,177,178,179,180].

Nutrigenomics is particularly relevant because diet can influence methyl-donor availability, one-carbon metabolism, acetyl-CoA and NAD^+^ pools, mitochondrial function, oxidative stress, inflammation, microbiome-derived signalling, and chromatin-modifying pathways [12,16,29,30,31,32,33,34]. Through these mechanisms, nutrition may affect DNA methylation, histone acetylation, non-coding RNAs, autophagy, nutrient sensing, and telomere-related stress responses [29,31,35,36,37,38]. However, these effects likely vary according to genetic differences in metabolism, inflammation, and nutrient-response pathways.

Geroscience has also expanded potential interventions beyond nutrition, including dietary restriction, fasting strategies, exercise, circadian alignment, senescence-targeted therapies, pharmacological geroprotectors, NAD^+^-related approaches, and microbiome or immune modulation. These interventions converge on pathways such as AMPK, mTOR, FOXO, sirtuins, autophagy, mitochondrial maintenance, NF-κB, NRF2, inflammatory signalling, and chromatin regulation [39,40,41,42]. Nevertheless, much evidence remains experimental, and human studies are limited by heterogeneity in phenotypes, tissues, biomarkers, and clinical interpretation [2,3,28,43,44,45,46,47,48,49,181,182,183,184,185,186,187,188,189,190,191,192,193,194,195,196,197,198,199,200,201,202,203,204,205,206,207,208,209,210,211,212,213,214,215,216,217,218,219,220,221,222,223,224,225,226,227,228,229,230,231,232,233,234,235,236,237,238,239,240,241,242,243,244,245,246,247,248,249,250,251,252,253,254,255,256,257,258,259,260,261,262,263,264,265,266,267,268,269,270,271,272,273,274,275,276,277,278,279,280,281,282,283,284,285,286,287,288,289,290,291,292,293,294,295,296,297,298,299,300].

The literature remains fragmented, as genetic, epigenetic, and intervention studies are often examined separately. This limits the understanding of how inherited susceptibility, epigenetic plasticity, nutritional modulation, and intervention responsiveness interact in ageing biology. Therefore, this review integrates three major dimensions: (i) genetic determinants of longevity, including telomeres, *FOXO3*, *APOE*, IGF1/insulin–mTOR signalling, sirtuins, Klotho, inflammation, and DNA repair; (ii) epigenetic mechanisms such as DNA methylation, epigenetic clocks, histone modifications, chromatin remodelling, non-coding RNAs, and links with telomere, mitochondrial, and immunometabolic dysfunction; and (iii) ageing-related interventions, including senescence-targeting therapies, geroprotectors, dietary and nutrigenomic strategies, exercise, circadian regulation, microbiome and immune-metabolic modulation, and biomarkers for precision response.

This review is important because it moves beyond isolated discussions of longevity genes, epigenetic biomarkers, or candidate anti-ageing interventions and instead frames ageing as a biologically integrated, environmentally responsive, and potentially stratifiable process. Its added value lies in explicitly linking nutrigenomics to both the molecular architecture of longevity and the epigenetic plasticity of ageing, while evaluating how these dimensions may shape responsiveness to interventions. By doing so, it highlights a key translational point: many interventions converge on shared molecular nodes, but their effects are likely to differ according to tissue context, baseline physiology, age, and inherited or acquired molecular profiles.

This narrative review was based on a structured search of PubMed, Scopus, Web of Science, and Embase, focusing mainly on recent English-language studies addressing genetic determinants of longevity, epigenetic mechanisms of ageing, and intervention-responsive pathways relevant to nutrigenomics and precision geroscience. Human studies were prioritized, while animal and in vitro studies were included when they provided important mechanistic insight. The literature was synthesized narratively to identify areas of mechanistic convergence, evidence limitations, and translational relevance. Grammarly (Platform Inc./Grammarly, San Francisco, CA, USA) was used only for superficial language editing. GPAI (GPAI: AI STEM CopilotVersion 1.3.0, Turing Co., Ltd., Seoul, South Korea) was used only for technical visual refinement of author-generated figures. No generative artificial intelligence tool was used to select, interpret, or generate scientific content.

Accordingly, the aim of this narrative review is to clarify how genetic susceptibility, epigenetic remodelling, and environmental or therapeutic modulation intersect in the biology of human ageing and longevity. By synthesizing these fields in a single framework, this review seeks to refine the rationale for diet-responsive ageing pathways, identify where human evidence is strongest or still limited, encourage more cautious interpretation of biomarkers and intervention claims, and support the development of precision nutrigeroscience, in which preventive and therapeutic strategies are better matched to inter-individual biological variability.

## 2. Genetic Determinants of Longevity

Human longevity is a polygenic, multifactorial trait shaped by genetic variation, environment, lifestyle, and stochastic processes. Its heritability is moderate, usually estimated at 12–25%, and depends on the phenotype analysed, such as lifespan, parental longevity, survival to old age, or exceptional longevity [1,2,3,4]. These outcomes overlap but should not be treated as equivalent in genetic studies. Most longevity-associated variants appear to have small effects and act through pathways related to cardiometabolic health, neurodegeneration, immunity, genome maintenance, stress resistance, and cellular resilience [1,2,3,5]. Exceptional longevity likely reflects both fewer deleterious alleles and enrichment of protective variants, with effects influenced by ancestry, sex, and phenotype definition [2,3,50,51]. Although GWAS have implicated loci involved in DNA repair, apoptosis, telomere biology, oxidative stress, lipid metabolism, cardiovascular function, and immune regulation, interpretation remains limited by phenotype heterogeneity, survivorship bias, ancestral imbalance, and proxy traits [2,3]. Overall, *FOXO3* and *APOE* remain the most consistently supported loci, while telomere maintenance, nutrient sensing, inflammation, and DNA repair pathways are biologically plausible but show more heterogeneous variant-level evidence.

### 2.1. Telomere Biology and Longevity

Telomere attrition is a recognized hallmark of ageing and a central mechanism linking replicative history to genome instability, cellular senescence, tissue dysfunction, and age-related disease [52,53,54,55,56,57,58]. Telomeres consist of tandem TTAGGG repeats bound by the shelterin complex, which protects chromosome ends from being recognized as DNA double-strand breaks. Because conventional DNA polymerases cannot fully replicate chromosome termini, telomeric DNA progressively shortens with each cell division through the end-replication problem [52,53,54,55,56,57,58,59,60]. When telomeres become critically short or structurally uncapped, shelterin protection is compromised, leading to activation of ATM/ATR-dependent DNA damage signalling, recruitment of DNA damage-response proteins, and induction of apoptosis or stable cell-cycle arrest [36,52,53,54,59,60,61,62,63,64,65,66,67,68,69,70,71,72,73]. Thus, telomere shortening is not only a marker of replicative history but also an active molecular trigger of senescence-associated cell fate decisions [36,63,64].

Telomere maintenance is counteracted by telomerase, a ribonucleoprotein complex composed of telomerase reverse transcriptase, the RNA template encoded by *TERC,* and accessory proteins required for assembly, trafficking, and telomere recruitment [53,54,56]. By extending telomeric repeats, telomerase preserves chromosome-end integrity and replicative potential [53,54,56]. However, telomere dysfunction is not determined by length alone. Defective shelterin-mediated capping can expose chromosome ends to DNA repair machinery and activate telomere dysfunction-induced foci even when telomere length remains within a nominally normal range [65]. Therefore, telomere biology involves at least three functionally distinct but interconnected components: telomere length, telomerase activity, and telomere-protective architecture.

At the cellular level, critically short or uncapped telomeres activate senescence primarily through the *TP53–CDKN1A*/p21 and *CDKN2A*/p16^INK4A^–RB1 pathways [74,75,76]. Persistent telomeric DNA damage signalling stabilizes p53, induces p21-mediated cell-cycle arrest, and may reinforce senescence through p16^INK4A^–RB1-dependent chromatin remodelling. Telomere dysfunction-induced senescence is also embedded within a broader stress network that includes oxidative stress, oncogene activation, mitochondrial damage, infection, nutrient imbalance, and inflammatory signalling [77,78,79]. Importantly, telomere-associated DNA damage foci may accumulate independently of measurable telomere shortening, including in post-mitotic cells, indicating that telomere-centred damage signalling contributes to ageing beyond simple replicative exhaustion [61,80].

Telomere biology also intersects mechanistically with nutrient sensing, mitochondrial function, epigenetic regulation, and inflammation. SIRT1 and SIRT6 are particularly relevant because they function as NAD^+^/NADH-sensitive chromatin and stress-response regulators [263,264,265]. SIRT1 deacetylates histone and non-histone substrates, including NF-κB, p53, PGC-1α, and Nrf2, thereby coordinating DNA repair, antioxidant defence, mitochondrial biogenesis, inflammatory tone, and genomic stability [266]. In telomere biology, SIRT1 has been implicated in delaying telomere shortening, promoting DNA repair, and regulating telomerase through modulation of *TERT* expression, nuclear localization, and protein stability, partly through the FOXO3a/c-MYC axis [267,268,269]. Conversely, telomere dysfunction can repress SIRT1 through p53-dependent microRNA-mediated mechanisms, suggesting a feedback loop in which telomere damage reduces SIRT1 activity, thereby further weakening mitochondrial, inflammatory, and chromatin maintenance programs [270]. SIRT6 also contributes to telomeric chromatin integrity and DNA repair, positioning sirtuin activity as a metabolic–epigenetic bridge between NAD^+^ availability, telomere protection, genome maintenance, and ageing-related stress responses [264,266,271,272,273,274,275,276,277,278,279,280,281].

In humans, interpretation remains more complex than in experimental systems. Most population studies use leukocyte telomere length, which is only a partial surrogate for telomere dynamics across tissues and may be influenced by immune-cell composition, inflammation, infection history, oxidative load, and proliferative turnover [35,36,52,53,54]. Moreover, longer telomeres are not uniformly beneficial. Short telomeres may promote chromosomal instability and tissue degeneration, but telomere shortening can also constrain malignant proliferation by limiting replicative potential [36,59,60]. Conversely, aberrant telomerase activation may support tumour progression, epithelial–mesenchymal transition, inflammatory remodelling, and expansion of mutant clones, including clonal haematopoiesis [293,294,295,296,297,298,299]. These trade-offs indicate that telomere length, telomerase activity, and telomere damage responses must be interpreted in relation to tissue context, proliferative history, cancer risk, and genomic background.

### 2.2. FOXO3 and Nutrient-Sensing Resilience Pathways

**Figure 1:**
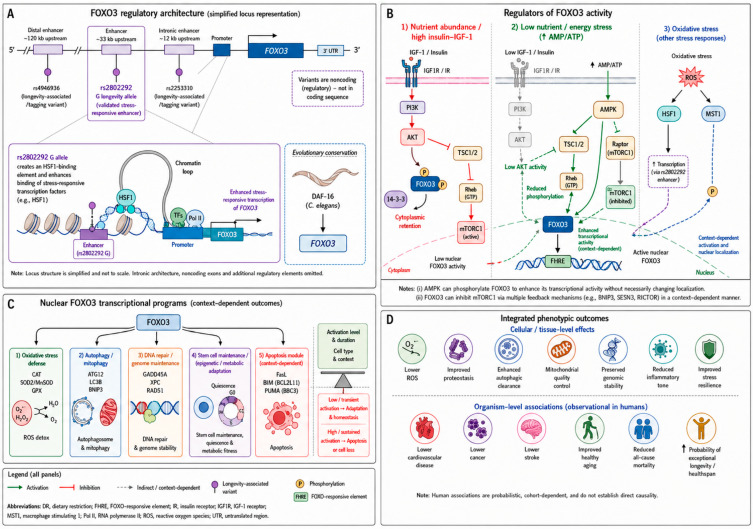
*FOXO3* regulatory architecture and signalling mechanisms underlying stress resilience and longevity-associated phenotypes. (**A**) Simplified representation of the *FOXO3* regulatory locus, including longevity-associated noncoding variants and the stress-responsive rs2802292 G allele. (**B**) Regulation of FOXO3 activity by nutrient abundance, low-energy conditions, and oxidative stress through insulin–IGF-1/PI3K/AKT, AMPK, HSF1, and MST1 signalling. (**C**) Context-dependent transcriptional programs activated by nuclear FOXO3, including oxidative-stress defense, autophagy, mitophagy, DNA repair, quiescence, metabolic adaptation, and apoptosis. (**D**) Integrated cellular effects and observational human associations linked to FOXO3 activity, including improved stress resilience, healthier aging, reduced disease burden, and increased probability of exceptional longevity. Note. GPAI: AI STEM Copilot (Turing Co., Ltd.) was used solely for the technical visual refinement of this author-generated figure. All scientific content, labels, and interpretations were created and verified by the authors.

Among candidate loci, *FOXO3* is one of the most consistently replicated associations in human longevity research, particularly in studies of exceptional longevity and survival in older adults [1,2,3,4,81]. The *FOXO3* gene encodes a transcription factor that binds FHRE consensus sequences and regulates genes involved in oxidative stress resistance, apoptosis, autophagy, DNA repair, metabolism, and stem cell maintenance, thereby supporting cellular homeostasis and stress adaptation [1,2,3,81]. The pathway is evolutionarily conserved; in *Caenorhabditis elegans*, the FOXO ortholog DAF-16 is a major mediator of lifespan regulation [2] (See Figure 1A–C).

The SNP rs2802292 is the most extensively characterized longevity-associated variant in *FOXO3*, with the minor G allele repeatedly associated with increased lifespan, reduced all-cause mortality, and higher probability of reaching extreme old age in several cohorts [1,2,3,4,82,83]. Some cohorts have reported an allele–dose relationship, although this pattern has not been assessed uniformly across all populations and study designs [1,4,82]. Additional variants, including rs2253310 and rs4946936, have also been associated with extended lifespan and survival in older adults [4]. Most longevity-associated *FOXO3* variants are noncoding and likely regulatory, suggesting effects on chromatin architecture, enhancer activity, or transcriptional responsiveness rather than protein structure; however, the causal alleles and their precise functional consequences remain incompletely defined [2,3] (See Figure 1A).

Mechanistically, FOXO3 acts as a downstream effector of the insulin/IGF-1/PI3K/AKT axis, linking nutrient availability to stress-response pathways [2,3,5,32,84]. Reduced IGF-1 signalling during dietary restriction is associated with decreased AKT activity, which relieves inhibitory phosphorylation of FOXO3 and promotes its nuclear activity, while concurrent inhibition of mTORC1 may further enhance stress resistance [2,5,84]. AMPK activation under energy stress can also activate FOXO3, suppress inflammatory signalling, and inhibit mTORC1, forming an integrated AMPK–FOXO3–mTOR axis relevant to resilience under oxidative and metabolic stress [2,84]. FOXO3 has also been reported to suppress mTORC1 via inhibition of Raptor, reinforcing reciprocal regulation between anabolic growth signalling and stress adaptation pathways [84] (See Figure 1B).

Consistent with these mechanisms, *FOXO3* variants have been associated with reduced risk of cardiovascular disease, cancer, stroke, and overall mortality, as well as improved self-reported health in elderly populations [2,3]. At the cellular level, FOXO3 promotes antioxidant defense through induction of catalase and MnSOD, regulates autophagy via targets such as ATG12 and BNIP3, and modulates apoptosis through genes including FasL, Bim, and Puma [84]. However, *FOXO3*-mediated signalling is context-dependent, and excessive activation may contribute to vascular instability or excessive apoptosis. *FOXO3* is therefore among the strongest human longevity loci, but the associated variants are largely regulatory, and their effects are not fully resolved (See Figure 1C,D).

### 2.3. APOE and Lipid-Mediated Determinants of Longevity

**Figure 2:**
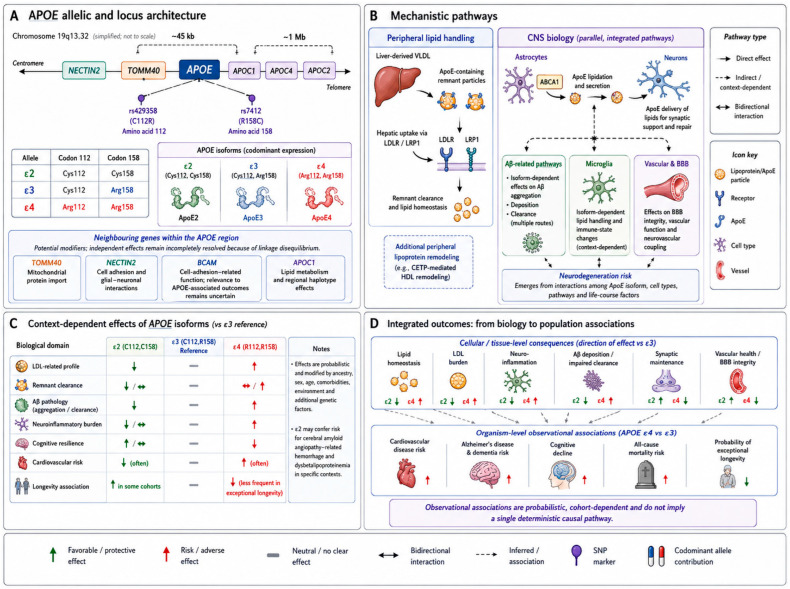
*APOE* allelic architecture, isoform-specific biology, and integrated associations with cardiometabolic and neurodegenerative outcomes. (**A**) Simplified representation of the chromosome 19q13.32 *APOE* region, including the rs429358 and rs7412 variants that define the ε2, ε3, and ε4 isoforms, together with neighbouring genes, the independent effects of which remain incompletely resolved because of linkage disequilibrium. (**B**) Overview of peripheral and central nervous system pathways influenced by ApoE, including remnant lipoprotein clearance, ABCA1-mediated ApoE lipidation, glial–neuronal lipid transport, Aβ-related processes, microglial responses, and vascular or blood–brain barrier function. (**C**) Context-dependent comparison of *APOE* isoforms relative to ε3, emphasizing the generally favourable but non-uniform profile of ε2 and the broader adverse associations of ε4 across lipid metabolism, neuroinflammatory burden, Aβ pathology, cardiovascular risk, and longevity-related traits. (**D**) Integrated cellular consequences and population-level observational associations linked to *APOE* ε4 relative to ε3, including increased cardiovascular and Alzheimer’s disease risk, cognitive decline, mortality risk, and reduced probability of exceptional longevity. Note. GPAI: AI STEM Copilot (Turing Co., Ltd.) was used solely for the technical visual refinement of this author-generated figure. All scientific content, labels, and interpretations were created and verified by the authors.

In addition to *FOXO3*, *APOE* is among the most consistently replicated loci associated with human longevity, although much of its effect appears to be mediated through age-related disease risk, especially neurodegeneration and cardiovascular disease [3,5]. The *APOE* gene, located on chromosome 19q33 within a cluster including *APOC1*, *APOC2*, *APOC4*, and *TOMM40*, encodes apolipoprotein E, which regulates cholesterol transport, neuronal repair, and immune responses [3,50,51,85]. Its three major alleles—ε2, ε3, and ε4—are defined by SNPs rs7412 and rs429358 and produce isoforms with distinct functional effects [3,50,85]. Thus, *APOE* is best interpreted in this review as a case of multiple allelism with codominant molecular expression and allele-specific phenotypic effects. The ε2, ε3, and ε4 alleles represent alternative allelic forms at the same locus; both inherited alleles can contribute to the expressed apolipoprotein E isoform profile, and their biological relevance to longevity arises from differential effects on lipid handling, neurodegeneration, cardiovascular risk, inflammation, and survival [50,85] (See Figure 2A).

The ε2 allele is enriched in centenarian-enriched cohorts and has been associated with lower LDL levels, reduced cardiovascular and neurodegenerative risk, improved cognitive function, and resistance to sarcopenia, although these associations are not identical across populations [3,5,50,51,81]. In contrast, ε4 is associated with increased risk of Alzheimer’s disease, cardiovascular disease, inflammatory burden, and reduced survival, although its effects may be modified by diet, lifestyle, sex, and ancestry [3,5,51,85]. The ε3 allele is the most common isoform and is generally used as the reference genotype in comparative analyses [3,51,85] (See Figure 2C,D).

Mechanistically, APOE influences longevity through regulation of lipid and cholesterol homeostasis, neuroinflammation, and gene-expression networks within the central nervous system [51,85]. *APOE* ε4 is associated with a more pro-inflammatory astrocytic phenotype and dysregulated microglial activation, thereby contributing to neurodegeneration, while interactions with neighbouring genes such as *TOMM40*, *NECTIN2*, and *BCAM* may further shape cognitive decline and brain ageing [85]. Epigenetic regulation adds further complexity, because *APOE* methylation patterns vary by brain region and genotype and may also respond to environmental exposures [51]. In many cohorts, the association between *APOE* and “longevity” likely reflects competing risks related to dementia and cardiovascular mortality rather than a single direct effect on ageing rate (See Figure 2A,B,D).

Other lipid-related genes, including *CETP*, have also been implicated in survival to advanced age, particularly in centenarian-enriched cohorts [3,5]. These associations are usually interpreted in the context of HDL remodelling, reverse cholesterol transport, and cardiometabolic risk, but findings differ across populations and cannot be reduced to quantity of HDL-cholesterol alone [3,5]. HDL function is likely more relevant than circulating HDL-C levels in isolation. Overall, lipid-handling pathways are important in longevity research, but *APOE* remains the clearest and most reproducible human signal within this group (See Figure 2B).

### 2.4. Nutrient Sensing, Genome Maintenance, and Inflammatory Resilience

In addition to the more consistently replicated longevity loci, particularly *FOXO3* and *APOE*, several interconnected pathways contribute to ageing by regulating nutrient sensing, metabolic adaptation, genome stability, endocrine function, and inflammation [6,7,8,9,10,11,12,13,14,15,16]. These include the IGF1/insulin axis, mTOR signalling, sirtuins, Klotho, IL-6-related inflammation, and DNA repair systems. Although these pathways are strongly supported by experimental and mechanistic ageing research, direct human variant-level evidence for longevity is generally less consistent than for *FOXO3* or *APOE* [6,7,8,9,10,11].

The IGF1/insulin–mTOR network links nutrient availability to growth, protein synthesis, metabolism, and tissue maintenance. IGF1 signalling appears to have age-dependent effects, supporting growth and repair earlier in life but potentially increasing mortality and cancer risk when chronically activated in later life [7]. Similarly, mTORC1 promotes anabolic metabolism and translation, while reduced mTOR activity and lower translational output are associated with improved proteostasis and lifespan extension in experimental models [6,11].

Sirtuins connect metabolic status with stress resistance, mitochondrial function, chromatin regulation, and DNA repair [8,9,10]. SIRT1 supports FOXO activity, mitochondrial biogenesis, vascular and metabolic health, and suppression of NF-κB-driven inflammation [81,82,84]. SIRT6 contributes to DNA repair, telomere maintenance, genomic stability, and chromatin organization [3,86], whereas SIRT7 regulates proteostasis, mitochondrial function, and stem cell maintenance, with context-dependent effects in ageing [8].

Klotho, encoded by *KL*, adds an endocrine and metabolic dimension to this network. It regulates mineral metabolism, oxidative stress, inflammation, and insulin/IGF-1 signalling [12]. Through activation of Nrf2 and inhibition of NF-κB, Klotho may reduce oxidative and inflammatory burden. *KL* polymorphisms such as G-395A, C1818T, and KL-VS have been associated with metabolic disease susceptibility, supporting its role as a plausible modifier of healthy ageing, although direct longevity associations remain less established [12].

Inflammatory and immune-regulatory mechanisms further shape ageing trajectories. Chronic low-grade inflammation, or inflammageing, involves mediators such as IL-6, TNF-α, and CRP and is driven by cumulative cellular damage and immune dysregulation [13,14,16,32]. *IL6* has been linked to inflammatory burden and mortality risk, but polymorphism data, circulating cytokine levels, and pathway activity should be interpreted as distinct forms of evidence [16,32]. Immune-metabolic pathways such as IDO1–kynurenine–AhR also connect inflammation with metabolic regulation and tissue degeneration [14].

Finally, DNA repair and genome maintenance preserve cellular function by limiting somatic mutation accumulation and maintaining chromatin integrity [15,16]. Failures in these systems are strongly linked to accelerated ageing, although common longevity-associated variants in these pathways usually show modest effects [15,16]. Sirtuins also contribute here by coordinating chromatin regulation, DNA repair, NF-κB inhibition, and immune responses [16].

Overall, these pathways should be understood as connected regulators of ageing resilience rather than isolated determinants of longevity. Their importance for nutrigenetics and nutrigenomics lies in their potential to explain why individuals differ in response to diet, energy balance, oxidative stress, inflammation, and geroprotective interventions. Therefore, even though their human variant-level evidence is weaker than that of *FOXO3* and *APOE*, they remain central to understanding biologically stratified ageing and personalized intervention responses [6,7,8,9,10,11,12,13,14,15,16].

## 3. Epigenetic Mechanisms in Ageing and Lifespan

Ageing is accompanied by a gradual decline in epigenetic maintenance and regulatory stability, commonly referred to as epigenetic drift. This includes cumulative changes in DNA methylation, histone modifications, chromatin organisation, and non-coding RNA regulation, which collectively contribute to transcriptional dysregulation, loss of cellular identity, reduced plasticity, genomic instability, and chronic low-grade inflammation. Because these changes may function as causal drivers, adaptive responses, downstream effects, or biomarkers depending on context, epigenetic ageing should be viewed as a regulatory framework rather than a single causal pathway. Its relevance to longevity research lies in its responsiveness to environmental and nutritional exposures and its potential contribution to inter-individual variation in ageing trajectories [17,18,19,20,21,22,23,24,25,26,27,28,87,88,89,90,91,92,93,94,95,96,97,98,99,100,101,102,103,104,105,106,107,108,109,110,111,112,113,114,115,116,117,118,119,120,121,122,123,124,125,126,127,128,129,130,131,132,133,134,135,136,137,138,139,140,141,142,143,144,145,146,147,148,149,150,151,152,153,154,155,156,157,158,159,160,161,162,163,164,165,166,167,168,169,170,171,172,173,174,175,176,177,178,179,180].

### 3.1. DNA Methylation Dynamics and Epigenetic Clocks

A widely observed feature of epigenetic ageing is the redistribution of DNAm patterns, characterised by global hypomethylation alongside site-specific hyper- and hypomethylation at regulatory genomic regions. These changes may arise, at least in part, from cumulative DNA damage and repair processes that progressively reshape the methylome. While some of these responses may initially be adaptive, chronic DNA repair-associated chromatin remodelling appears to disrupt epigenetic homeostasis, contributing to epigenetic drift and reduced stability of cell-type-specific transcriptional programs [17,87].

Importantly, age-associated DNAm changes are highly structured rather than random, providing the molecular basis for epigenetic clocks. These predictive models estimate chronological age from methylation levels at defined subsets of CpG sites and remain among the most robust biomarkers in ageing research [18]. Epigenetic age acceleration derived from these models is associated with morbidity and mortality risk. However, because clocks are trained predictors built from selected CpG loci, they should be interpreted as composite molecular readouts, not as direct measures of a single biological ageing process [18,23,24]. Different clocks capture partly distinct methylation features, risk-related dimensions, and, in some cases, cell-composition effects.

Different classes of epigenetic clocks, therefore, reflect complementary aspects of ageing biology. Multi-tissue clocks tend to capture more conserved, cell-intrinsic DNAm changes, whereas tissue-specific clocks also incorporate contextual signals, including local cellular composition and microenvironmental influences [17,27]. More recent cell-type-resolved and single-cell approaches further indicate that ageing trajectories differ substantially across individual cell populations, revealing marked intra-tissue heterogeneity [25,26]. Consistent with this, epigenetic ageing varies across organs: tissue-specific clocks may better reflect local functional states, whereas blood-based measures correlate only imperfectly with other tissues and are therefore limited as proxies for whole-body physiological ageing [28].

From a nutrigenomic perspective, DNAm is especially relevant because methyl-group availability, one-carbon metabolism, inflammatory tone, and metabolic status may all influence methylation maintenance and remodelling. Genetic variability in nutrient handling and methyl-donor pathways may further condition the magnitude and direction of these responses. Thus, DNAm ageing links nutrition, genotype, and regulatory plasticity, although causality remains dependent on tissue, exposure history, and cell composition.

### 3.2. Genomic Patterning of Age-Associated DNA Methylation

The ageing methylome follows reproducible genomic patterns, suggesting a dual regulatory architecture in which conserved alterations coexist with tissue-specific changes that shape gene expression in a context-dependent manner.

Hypermethylation preferentially affects CpG-rich promoters of developmental genes located within Polycomb-repressed and bivalent chromatin domains. These regions are normally maintained in a poised state, enabling rapid activation during development, repair, or regeneration. With ageing, hypermethylation at such loci is thought to reinforce transcriptional repression, which may restrict cellular plasticity and impair regenerative potential [17,88]. Polycomb group (PcG) proteins mediate this regulation through two major complexes: Polycomb Repressive Complex 2 (PRC2), which deposits the repressive H3K27me3 mark, and Polycomb Repressive Complex 1 (PRC1), which stabilises gene silencing through histone ubiquitination. Multiple PRC variants may contribute to selective targeting of genomic regions and thus to the structured nature of age-associated epigenetic repression [89,90]. These observations support a role for reinforced Polycomb-mediated silencing in senescence and diminished tissue regeneration, while leaving open whether methylation changes are causal or reflect altered cell state [17].

In contrast, hypomethylation tends to occur in CpG-poor intronic and intergenic regions enriched for active enhancer elements. Because these regions are associated with activating histone marks and transcriptional activity, enhancer hypomethylation may contribute to tissue-specific transcriptional dysregulation during ageing [91]. Some studies have proposed that this pattern reflects reduced fidelity of DNMT and TET enzyme activity, although the upstream drivers remain incompletely defined and may vary across tissues. These alterations may perturb enhancer–promoter interactions and co-methylation networks, thereby compromising transcriptional precision. Collectively, redistribution of DNAm across promoters and enhancers is consistent with the broader loss of transcriptional fidelity and cellular homeostasis observed with ageing [19].

This dimension of ageing biology is also relevant to nutrition, since methyl-donor supply and metabolic cofactors linked to one-carbon metabolism can influence DNAm maintenance. However, tissue specificity and cell-type composition remain central interpretive constraints, particularly in blood-based human studies.

### 3.3. Regulation of Histone Modifications, Chromatin State, and Metabolic–Epigenetic Coupling

**Figure 3:**
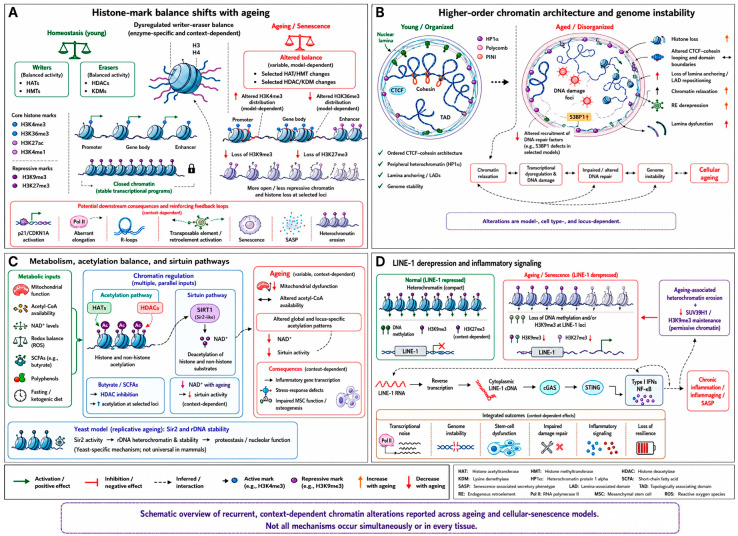
Chromatin remodelling, genome instability, and inflammatory signalling during ageing and cellular senescence (**A**). Ageing and senescence disrupt the balance between histone-mark writers and erasers, leading to altered activating and repressive histone modifications. These changes promote chromatin relaxation, transcriptional dysregulation, retroelement activation, and heterochromatin erosion. (**B**) Young cells maintain organized chromatin loops, lamina anchoring, and stable topologically associating domains. During ageing, chromatin architecture becomes disorganized, contributing to DNA damage, altered repair responses, genome instability, and cellular ageing. (**C**) Metabolic changes associated with ageing, including altered acetyl-CoA availability, reduced NAD^+^ levels, and mitochondrial dysfunction, affect histone acetylation and sirtuin activity. These changes influence inflammatory gene expression, stress responses, and stem-cell function. (**D**) Loss of heterochromatin at LINE-1 loci promotes retroelement transcription and cytoplasmic DNA accumulation. Activation of the cGAS–STING pathway then drives type I interferon signalling, chronic inflammation, and the senescence-associated secretory phenotype. Note. GPAI: AI STEM Copilot (Turing Co., Ltd.) was used solely for the technical visual refinement of this author-generated figure. All scientific content, labels, and interpretations were created and verified by the authors.

Histone modifications and chromatin remodelling constitute tightly connected mechanisms through which ageing reshapes transcriptional regulation, genome stability, and inflammatory signalling. Histone marks, including methylation, acetylation, and phosphorylation, are dynamically regulated by “writer” enzymes, such as HATs and HMTs, and “eraser” enzymes, such as HDACs and KDMs [20,21]. During ageing, disruption of this balance alters chromatin accessibility, weakens transcriptional fidelity, destabilizes heterochromatin, and facilitates inflammatory gene activation [92]. These changes do not occur in isolation but interact with higher-order chromatin reorganization, nuclear lamina dysfunction, repetitive-element derepression, and DNA damage accumulation [20,22] (See Figure 3A,B,D).

A central feature of epigenetic ageing is the simultaneous dysregulation of activating marks and erosion of repressive chromatin. H3K4me3, normally enriched at transcription start sites and associated with active promoters, may become excessive or mislocalized, promoting *CDKN1A*/p21 activation, aberrant transcriptional elongation, R-loop formation, and genomic instability in stem-cell and stress models [93,94]. Altered H3K36me3, which normally preserves transcriptional fidelity across gene bodies, has been linked to endogenous retrovirus activation and premature senescence [95,96]. In parallel, loss of repressive marks such as H3K9me3 and H3K27me3 compromises constitutive and facultative heterochromatin, increasing chromatin accessibility at normally silenced regions, including developmental loci and repetitive elements [22,98,99,114,115]. Senescence-associated KDM4 upregulation further reduces H3K9 and H3K36 methylation and may amplify the senescence-associated secretory phenotype [97] (See Figure 3A).

These histone-level changes are reinforced by large-scale disruption of chromatin architecture. Ageing is associated with histone loss, altered chromatin looping, impaired CTCF-associated genome organization, and weakening of heterochromatin regulators such as HP1a, Polycomb proteins, and PIN1 [20,114]. Nuclear lamina dysfunction further destabilizes genome organization by impairing heterochromatin anchoring at the nuclear periphery. This mislocalization may reduce the recruitment of DNA repair factors, including BRCA1 and 53BP1, while increased chromatin accessibility exposes DNA to damaging stressors, creating a self-reinforcing cycle of chromatin relaxation, DNA damage accumulation, defective repair, and cellular ageing [114,116] (See Figure 3B).

Histone acetylation provides a direct mechanistic bridge between chromatin state and metabolism. By neutralising lysine charge, acetylation generally increases chromatin accessibility and transcriptional activation [29]. Ageing has been associated with a “global loss–local gain” acetylation pattern, characterized by reduced overall acetylation but increased acetylation at selected loci, particularly inflammatory genes [100]. Reduced enhancer acetylation may impair mesenchymal stem-cell osteogenesis and proliferation, whereas locus-specific increases in H3K27ac and H3K4me1 have been linked to HMGB2-dependent transcriptional activation and partial rejuvenation-like effects in senescent mesenchymal stem cells [37,38]. Thus, acetylation is not uniformly protective or detrimental; its consequences depend on genomic location, cell type, tissue state, and stress context (See Figure 3C).

Mechanistically, age-related acetylation changes reflect the coupling between chromatin-modifying enzymes, mitochondrial function, acetyl-CoA availability, NAD^+^-dependent sirtuin activity, and redox balance [30,31]. Reduced mitochondrial function may limit acetyl-CoA availability and contribute to global hypoacetylation, whereas NAD^+^ decline may impair sirtuin-dependent deacetylation, linking metabolic deterioration to inflammatory transcription, defective stress responses, and chromatin instability [30,31]. Similar metabolic–epigenetic coupling is evident at ribosomal DNA regions, where reduced histone acetylation and decreased Sir2 activity may compromise rDNA stability, proteostasis, and nucleolar function during ageing [117] (See Figure 3C).

One major downstream consequence of heterochromatin failure is reactivation of normally silenced repetitive elements, particularly LINE-1 retrotransposons. Under physiological conditions, LINE-1 elements are suppressed by DNA methylation and repressive histone marks. During ageing, global hypomethylation together with H3K9me3 and H3K27me3 depletion weakens this repression, allowing LINE-1 transcriptional reactivation [118]. In senescent cells, LINE-1 RNA can undergo reverse transcription, generating cytoplasmic DNA intermediates that activate the cGAS-STING pathway, induce type I interferon responses, and promote chronic inflammation [119,120]. LINE-1 activation may also reinforce epigenetic instability by interfering with heterochromatin regulators such as Suv39H1, thereby aggravating H3K9me3 loss and chromatin erosion [98,99] (See Figure 3D).

From a nutrigenomic perspective, these mechanisms are particularly relevant because chromatin maintenance is sensitive to metabolite availability, mitochondrial status, inflammatory tone, and redox balance. Dietary and metabolic inputs, including short-chain fatty acids such as butyrate, polyphenol-responsive pathways, fasting or ketogenic states, acetyl-CoA availability, and NAD^+^-dependent sirtuin activity, may influence histone acetylation, deacetylation, heterochromatin stability, and transposable-element repression [30,31]. Genetic variation in nutrient-sensing, inflammatory, antioxidant, and chromatin-regulatory pathways may further contribute to interindividual differences in epigenetic ageing and nutrigenomic response (See Figure 3C,D).

### 3.4. Non-Coding RNAs in the Regulation of Ageing

**Figure 4:**
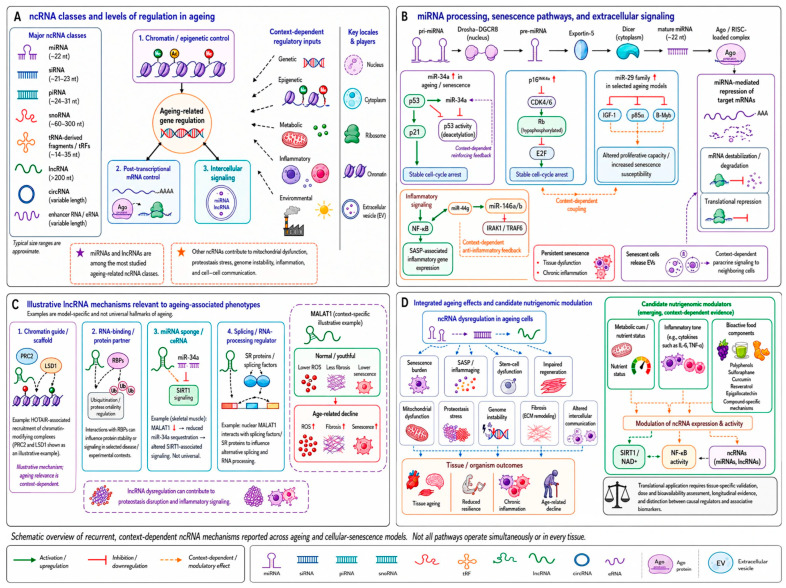
Non-coding RNA regulation in ageing: molecular mechanisms, senescence pathways, and nutrigenomic modulation. (**A**) Overview of the major classes of non-coding RNAs and their principal levels of action in ageing-related gene regulation, including chromatin control, post-transcriptional regulation, and intercellular signalling. (**B**) Schematic representation of miRNA biogenesis and selected miRNA-mediated pathways involved in cellular senescence, inflammatory signalling, mRNA repression, and extracellular vesicle communication. (**C**) Illustrative mechanisms through which lncRNAs may influence ageing-associated phenotypes, including chromatin remodelling, RNA-binding protein interactions, miRNA sponging, and RNA-processing regulation. (**D**) Integrated model of ncRNA dysregulation in ageing cells and its contribution to senescence, inflammaging, mitochondrial dysfunction, impaired regeneration, and tissue decline, together with potential nutrigenomic modulators. Note. GPAI: AI STEM Copilot (Turing Co., Ltd.) was used solely for the technical visual refinement of this author-generated figure. All scientific content, labels, and interpretations were created and verified by the authors.

Non-coding RNAs modulate ageing-related gene expression programs by linking chromatin state, mRNA stability, and intercellular signalling. They therefore add an important regulatory layer to ageing biology rather than constituting a single unified mechanism. Non-coding RNAs (ncRNAs) comprise a heterogeneous group of RNA molecules that are not translated into proteins but regulate gene expression at transcriptional, post-transcriptional, and epigenetic levels. The major ncRNA classes include small ncRNAs, such as microRNAs (miRNAs), small interfering RNAs, Piwi-interacting RNAs, small nucleolar RNAs, and transfer RNA-derived fragments, as well as long non-coding RNAs (lncRNAs), circular RNAs, and enhancer RNAs [101,102,103,110,111]. These classes differ in size, biogenesis, localization, and mechanism of action. In ageing, miRNAs and lncRNAs are the most extensively studied, but other ncRNAs are increasingly linked to cellular senescence, mitochondrial dysfunction, proteostasis, genomic instability, inflammatory signalling, and intercellular communication [101,102,103,104,108,109,110,111]. Mechanistically, ageing-related ncRNAs may regulate mRNA degradation and translation, recruit chromatin-modifying complexes, act as molecular scaffolds or decoys, modulate RNA splicing and stability, sponge miRNAs, influence transposable element activity, and participate in extracellular vesicle-mediated signalling [101,102,103,108,110,111,112,113]. Thus, ncRNAs provide a flexible regulatory interface through which genetic, epigenetic, metabolic, inflammatory, and environmental signals can be integrated during ageing (See Figure 4A–C).

MicroRNAs (miRNAs) are small non-coding RNAs of approximately 22 nucleotides that regulate gene expression post-transcriptionally through mRNA degradation or translational repression. They are implicated in cellular senescence, a major hallmark of ageing [101,102]. In human systems, miRNAs modulate core senescence-associated pathways, including the p53/p21 and p16/Rb axes, as well as the SASP, thereby influencing both cell-autonomous and non-cell-autonomous ageing processes [101,103]. Senescence, initially characterised in vitro and now widely documented in vivo, can act as a tumour-suppressive mechanism but may also contribute to tissue dysfunction and chronic inflammation through persistent SASP signalling [104] (See Figure 4B).

At the molecular level, several miRNAs converge on central senescence pathways. For example, miR-34a promotes senescence by repressing SIRT1, thereby enhancing p53 activity and favouring senescence-associated phenotypes [101,105]. Members of the miR-29 family have likewise been associated with senescence and impaired regenerative capacity in vivo through p53-related signalling [101,106]. By contrast, miR-146a modulates inflammatory signalling and SASP-related pathways through effects on NF-κB and NAD^+^/SIRT signalling, although its actions appear context dependent [107]. In addition to intracellular effects, senescent cells secrete extracellular vesicles enriched in miRNAs, potentially propagating senescence-associated signals to neighbouring cells and thereby contributing to tissue-level ageing phenotypes [108]. Current evidence therefore supports miRNAs as modulators of senescence-associated networks, while their role as primary drivers of organismal ageing remains unresolved [109] (See Figure 4B).

Long non-coding RNAs (lncRNAs) further refine ageing-related regulation through tissue-specific and context-dependent mechanisms. Age-associated lncRNAs (age-lncRNAs) are heterogeneous but are often co-expressed with protein-coding genes involved in immune responses, transcriptional regulation, RNA processing, proteostasis, and metabolism [103,110,111]. This pattern suggests that lncRNAs may fine-tune ageing-related pathways rather than globally control them. Mechanistically, lncRNAs interact with chromatin modifiers, RNA-binding proteins, and miRNAs. *HOTAIR*, for example, modulates proteostasis and inflammatory signalling by interacting with RNA-binding proteins and E3 ubiquitin ligases, promoting ubiquitination and degradation of target proteins while sustaining NF-κB activity during DNA damage and senescence [112]. *MALAT1* affects RNA processing and post-transcriptional regulation by interacting with splicing factors and functioning as a competing endogenous RNA. Age-related declines in *MALAT1* expression have been associated with enhanced oxidative stress responses, fibrosis, and cellular senescence, partly through altered availability of senescence-associated miRNAs such as miR-34a [113] (See Figure 4C).

From a nutrigenomic standpoint, ncRNA networks are plausible mediators of diet-responsive ageing effects, since inflammatory status, metabolic cues, and bioactive food components may influence miRNA and lncRNA expression. Their translational use, however, will require stronger tissue-specific validation and clearer distinction between causal regulators and downstream transcriptional markers (See Figure 4D).

### 3.5. Intersections with Telomere, Mitochondrial, and Immunometabolic Ageing

Epigenetic ageing intersects with other hallmarks of ageing, particularly telomere attrition, mitochondrial dysfunction, and immunometabolic decline. These links are relevant to lifespan regulation, but they are best viewed as points of convergence with epigenetic ageing rather than epigenetic mechanisms themselves.

A well-studied example is the relationship between telomere dysfunction and mitochondrial homeostasis. Telomere shortening and mitochondrial dysfunction appear to interact bidirectionally in a cycle associated with cellular senescence and organismal ageing [35,44]. Telomere dysfunction activates p53, which represses PGC-1α and PGC-1β, key regulators of mitochondrial biogenesis and oxidative metabolism [35,45,121]. This axis has been linked to reduced mitochondrial biogenesis, impaired oxidative phosphorylation, lower ATP production, and accumulation of dysfunctional mitochondria [36,45,121]. In parallel, dysfunctional mitochondria generate reactive oxygen species that may exacerbate telomeric damage and accelerate telomere loss [35,44,122]. Telomeres also have heterochromatin-like features, including dependence on H3K9me3, H4K20me3, and HP1α-associated organisation, supporting the view that telomere erosion is also a chromatin-associated event [123,124,125,126,127]. Additional work on TERT suggests that telomerase biology in ageing may involve not only telomere maintenance but also mitochondrial protection and redox regulation [128,129,130,131,132,133,134].

These intersections are also evident in immune ageing and obesity. In immune cells, progressive telomere shortening together with declining telomerase activity is widely regarded as a feature of immunosenescence, although interpretation of leukocyte telomere length is complicated by cell turnover, inflammation, sex differences, and disease burden [135,136,137,138,139,140,141]. Persistent infections and chronic metabolic stress have been associated with accelerated telomere erosion, while senescent T-cell populations show reduced proliferative capacity, altered telomerase activity, and increased stress signalling [61,142,143,144,145,146,147,148,149,150,151]. Obesity provides another context in which epigenetic, telomeric, and metabolic ageing converge, sharing features such as chronic inflammation, mitochondrial dysfunction, impaired nutrient sensing, cellular senescence, hypomethylation, and telomere attrition [152,153,154,155,156,157,158,159]. Long-term obesity has been associated with accelerated epigenetic ageing and telomere shortening, alongside inflammatory activation and cardiometabolic dysfunction [160,161].

For longevity, nutrigenetics, and nutrigenomics, the main implication is that epigenetic ageing is embedded within broader metabolic and inflammatory systems that are themselves sensitive to nutritional exposure. Diet-related effects on mitochondrial function, oxidative stress, NAD^+^ metabolism, immune activation, adiposity, and one-carbon metabolism may therefore influence biological ageing both indirectly and through direct chromatin effects. Genetic variation is likely to modify these responses, making this intersection central to precision nutrigeroscience.

### 3.6. Stress-Responsive Epigenetic Pathways Relevant to Ageing and Longevity

Psychosocial stress illustrates how non-nutritional environmental exposures may become biologically embedded through epigenetic mechanisms relevant to ageing, while remaining peripheral to the main focus on genetics and nutrigenomics in this review. Human studies have linked stress exposure to altered DNA methylation and histone regulation in pathways involving glucocorticoid signalling, neuroplasticity, serotonergic regulation, inflammation, DNA repair, apoptosis, and metabolism [49,162,163,164,165,166,167,168,169,170,171,172,173,174,175,176]. Key examples include *NR3C1* and *FKBP5*, which regulate HPA-axis feedback and glucocorticoid sensitivity; *BDNF*, which influences neuroplasticity and stress-related neuronal adaptation; and *SLC6A4*, which contributes to serotonergic signalling and cortisol reactivity [162,163,164,165,166,167,168,169,170,171].

Mechanistically, increased *NR3C1* methylation may reduce glucocorticoid receptor expression and feedback sensitivity, whereas altered *FKBP5* methylation may favour glucocorticoid resistance and prolonged stress signalling [167,168,169]. Stress-related methylation or reduced histone acetylation at *BDNF* promoters may impair neuroplasticity, while epigenetic regulation of *SLC6A4* may influence transporter expression and stress-responsive neural circuitry [165,166,170,171]. These changes may intersect with ageing through chronic inflammation, metabolic dysregulation, impaired DNA repair, and reduced stress resilience [163,172,177,178,179,180].

However, stress-related epigenetic signatures are highly context-dependent and vary by tissue, developmental timing, exposure intensity, genotype, disease state, and cell-type composition. Most human evidence remains observational and variably replicated [49,164,174,175,176]. Therefore, psychosocial stress is best framed as an example of gene–environment interaction and epigenetic plasticity rather than as a core longevity pathway or uniform biomarker of biological ageing. Its relevance lies in showing how environmental exposures may indirectly shape ageing trajectories through neuroendocrine, inflammatory, metabolic, and stress-response networks.

Overall, age-related epigenetic changes converge on reduced transcriptional precision, impaired cellular plasticity, heterochromatin erosion, repetitive-element derepression, inflammation, and diminished stress resilience. These mechanisms provide a plausible framework through which nutrition may influence biological ageing, while genetic variation may condition individual responses. However, many mechanistic links remain stronger in experimental systems than in human intervention studies, and biomarker modulation should not be equated with clinically meaningful slowing of organismal ageing (See Figure 2).

## 4. Gene–Environment Modulation of Ageing: Molecular Targets, Biomarkers, and Precision Response

Ageing is now understood as a biologically regulated and modifiable process influenced by genetic, environmental, and lifestyle factors. It involves interconnected changes in gene expression, signalling pathways, inflammation, nutrient sensing, mitochondrial function, autophagy, and stress responses. Since interventions such as diet, exercise, circadian regulation, pharmacological agents, and senescence-targeting therapies affect overlapping pathways but produce variable outcomes, precision geroscience is needed to tailor strategies to specific tissues, ageing mechanisms, and individual molecular profiles [2,3,28,43,44,45,46,47,48,49,181,182,183,184,185,186,187,188,189,190,191,192,193,194,195,196,197,198,199,200,201,202,203,204,205,206,207,208,209,210,211,212,213,214,215,216,217,218,219,220,221,222,223,224,225,226,227,228,229,230,231,232,233,234,235,236,237,238,239,240,241,242,243,244,245,246,247,248,249,250,251,252,253,254,255,256,257,258,259,260,261,262,263,264,265,266,267,268,269,270,271,272,273,274,275,276,277,278,279,280,281,282,283,284,285,286,287,288,289,290,291,292,293,294,295,296,297,298,299,300].

### 4.1. Pharmacological Targeting of Ageing Pathways

**Figure 5:**
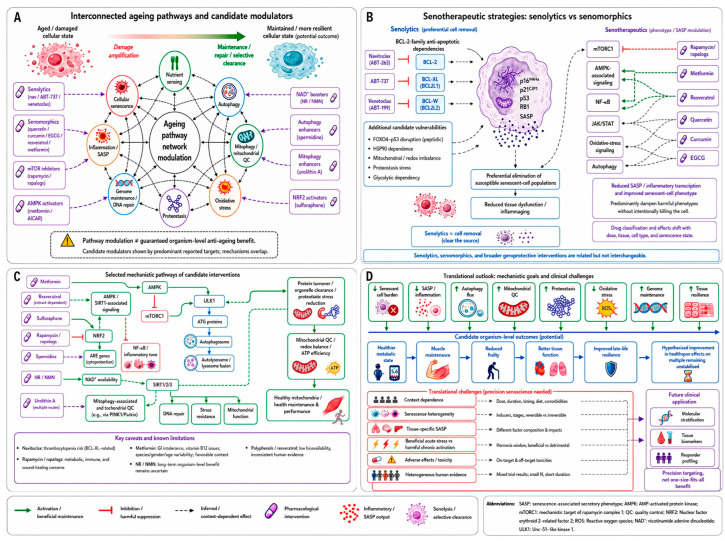
Interconnected Mechanisms of Ageing and Emerging Therapeutic Strategies: From Cellular Pathways to Clinical Translation. (**A**) Schematic overview of the interconnected biological processes involved in ageing, including cellular senescence, inflammation, nutrient sensing, autophagy, mitochondrial quality control, oxidative stress, proteostasis, and genome maintenance. Candidate interventions are positioned according to their predominant reported targets. (**B**) Comparison of senotherapeutic approaches. Senolytics aim to preferentially eliminate susceptible senescent cells, whereas senomorphics modulate the senescence-associated secretory phenotype and inflammatory signalling without necessarily inducing cell death. (**C**) Summary of selected mechanistic pathways through which candidate interventions may act. The diagram highlights the relationships between compounds such as metformin, resveratrol, rapamycin, spermidine, NAD^+^ boosters, sulforaphane, and urolithin A and pathways involving AMPK, mTORC1, NRF2, SIRT proteins, autophagy, mitophagy, and mitochondrial function. (**D**) Translational perspective linking pathway modulation to potential organism-level outcomes, including improved metabolic health, muscle maintenance, reduced frailty, better tissue function, and increased resilience. The panel also emphasizes major clinical challenges, such as context dependence, senescence heterogeneity, tissue-specific effects, toxicity, and variability in human evidence. Note. GPAI: AI STEM Copilot (Turing Co., Ltd.) was used solely for the technical visual refinement of this author-generated figure. All scientific content, labels, and interpretations were created and verified by the authors.

Pharmacological strategies in geroscience aim to modulate conserved ageing pathways rather than treat ageing as a single molecular lesion. Most candidate interventions converge on interconnected networks controlling cellular senescence, nutrient sensing, autophagy, mitochondrial quality control, oxidative stress, inflammation, proteostasis, and genome maintenance [39,40]. These pathways act as regulatory hubs through which pharmacological agents may shift cells from damage-amplifying states toward maintenance, repair, or selective clearance. However, their translational interpretation requires caution, because pathway modulation does not necessarily equate to clinically meaningful slowing of organismal ageing (See Figure 5A,D).

Cellular senescence is a major therapeutic target because senescent cells remain metabolically active, resist apoptosis, and secrete pro-inflammatory mediators through the senescence-associated secretory phenotype (SASP) [39,181,182]. Senescence is maintained by *CDKN2A*/p16^INK4a^, *CDKN1A*/p21^CIP1^, *TP53*/p53, and *RB1*/RB axes, together with chromatin remodelling and transcriptional rewiring [39]. Although transient senescence contributes to tumour suppression and tissue repair, chronic senescent-cell accumulation promotes tissue dysfunction, matrix remodelling, and inflammageing through cytokines and proteases such as IL-6, IL-8, TNF-α, and matrix-remodelling enzymes [39,42,182] (See Figure 5A,B).

Pharmacological approaches to senescence either eliminate senescent cells or suppress their deleterious secretory output. Senolytic activity exploits the dependence of senescent cells on pro-survival pathways, including BCL2-family members such as BCL2, BCL2L1/BCL-XL, and BCL2L2/BCL-W [39,183,184]. Agents such as navitoclax, ABT-737, and venetoclax target these anti-apoptotic dependencies in experimental models [39,185,186]. Other strategies attempt to restore apoptotic competence by disrupting FOXO4–TP53 interactions, inhibiting HSP90-dependent senescence signalling, or targeting vulnerabilities linked to mitochondrial dysfunction, redox imbalance, proteostasis stress, and altered glycolytic metabolism [187,188,189,190,191,192,193]. Natural compounds such as fisetin and piperlongumine have also been reported to influence apoptosis-, redox-, and stress-response programs, although their mechanisms and translational relevance remain context-dependent [194,195,196] (See Figure 5B).

In parallel, senomorphic interventions aim to restrain SASP signalling without necessarily removing senescent cells. Compounds such as quercetin, curcumin, EGCG, resveratrol, rapamycin, and metformin can attenuate inflammatory transcriptional programs by modulating NF-κB, JAK/STAT, mTOR, AMPK, oxidative stress, and autophagy-linked pathways [197,198,199,200]. This distinction between senolytics and senomorphics is useful conceptually but not absolutely, because several agents may shift between cytostatic, anti-inflammatory, and pro-apoptotic effects depending on dose, exposure duration, cell type, and senescence state [201,202] (See Figure 5B).

Many of the same pharmacological candidates also act through nutrient-sensing and maintenance pathways, particularly the AMPK–mTOR axis. Metformin activates AMPK and suppresses mTOR-associated anabolic signalling, thereby influencing autophagy, mitochondrial oxidative stress, inflammatory regulation, and NF-κB activity [200,203,204]. Although animal studies link metformin to improved metabolic function and lifespan-related outcomes [205,206,207,208], human evidence remains difficult to interpret because benefits in diabetic populations may reflect disease-specific metabolic effects rather than direct geroprotection [43]. Associations with epigenetic ageing markers are also inconsistent [209,210]. Rapamycin has stronger preclinical support: by inhibiting mTORC1, it reduces translation-intensive growth programs and promotes autophagy-dependent maintenance, with lifespan effects reproduced across several model organisms, including late-life treatment in mice [40,43]. These effects involve autophagy-related genes such as *ATG1*, *ATG5*, and *ATG7* and may also reduce SASP amplification through decreased IL1A translation [39,211,212,213]. However, adverse effects such as insulin resistance, dyslipidaemia, and impaired wound healing constrain clinical translation [40,43] (See Figure 5B,C).

Other interventions target mitochondrial quality control, NAD^+^-dependent regulation, and cytoprotective stress responses. Spermidine promotes autophagy through mechanisms requiring intact autophagic machinery [214,215]. NAD^+^ precursors such as nicotinamide riboside may influence mitochondrial function, sirtuin activity, DNA repair, stress resistance, and inflammatory signalling [216,217,218]. Urolithin A promotes mitophagy and may support muscle function through mitochondrial quality-control pathways [219,220]. Resveratrol affects SIRT1, AMPK, and oxidative stress-response pathways, but human evidence remains inconsistent and limited by bioavailability [43,221]. Sulforaphane may activate cytoprotective responses through NFE2L2/NRF2-dependent signalling [222,223] (See Figure 5C).

Overall, pharmacological geroprotection is best understood as network modulation across senescence, nutrient sensing, autophagy, mitochondrial maintenance, inflammation, and stress resistance, rather than as discrete drug-class effects. The strongest experimental evidence currently supports mTOR inhibition, autophagy-linked maintenance, and selected senescence-targeted strategies, whereas human evidence remains heterogeneous for many candidates. A major translational challenge is that ageing-related pathways are context-dependent: senescence is molecularly diverse, SASP composition varies across tissues, and beneficial stress-response or repair programs may become harmful when chronically activated. Therefore, future applications will likely require molecular stratification, tissue-specific biomarkers, and responder profiling rather than assumptions of universal geroprotective benefit [39,40,43] (See Figure 5A,D).

### 4.2. Dietary Restriction and Nutritional Interventions: A Nutrigenomic Perspective

Dietary restriction (DR) remains the most robust non-pharmacological intervention in experimental ageing biology, but it includes distinct strategies rather than a single intervention. Caloric restriction, intermittent fasting, and time-restricted feeding differ in nutrient quantity, nutrient timing, and, in some cases, downstream physiology [33]. In a nutrigenomic context, these interventions are especially important because they illustrate how environmental inputs regulate longevity through gene expression, chromatin remodelling, metabolic signalling, and epigenetic plasticity. Across model organisms, DR consistently extends lifespan and delays age-related disease, whereas in humans, it more reliably improves healthspan-related traits and biological ageing markers than maximal lifespan itself [33,43].

Mechanistically, dietary restriction shifts cellular programs away from anabolic growth and toward repair and maintenance. This includes suppression of insulin/IGF-1 and mTOR signalling, activation of AMPK and sirtuin-related pathways, and induction of autophagy [34,43]. In lower organisms, the longevity effects of DR require core autophagy genes such as *ATG5*, *ATG7*, and *BECN1*, indicating that the response is genetically mediated rather than merely physiological [224,225,226]. Human studies suggesting increased expression of autophagy-related genes such as *BECN1* and *LC3B* under long-term caloric restriction support the view that dietary interventions act through regulated maintenance pathways [227]. Time-restricted feeding adds another layer by aligning nutrient exposure with circadian programs, implying that feeding time can influence metabolic regulation beyond caloric load alone [228].

These interventions should not be treated as interchangeable. Caloric restriction primarily alters nutrient quantity and energy availability; intermittent fasting changes patterns of fasting and refeeding; time-restricted feeding emphasizes timing and circadian alignment. Their effects may also depend on dietary composition. Mediterranean-style and plant-rich diets may influence inflammatory, oxidative, and metabolic pathways through polyphenols, unsaturated fatty acids, fiber-derived microbial metabolites, and methyl-donor availability [229,230]. This broadens the discussion from energy restriction alone to gene–environment interactions shaped by nutrient quality, timing, and composition.

Response heterogeneity is likely substantial. The molecular response to dietary interventions may depend on genetic variation in nutrient-sensing pathways, baseline metabolic phenotype, sex, age, microbiome composition, and population-level differences in metabolic response. One-carbon metabolism and methyl-donor availability may also be relevant because they connect nutrition to epigenetic plasticity. These sources of variability make dietary intervention a central model for nutrigenomics rather than a uniform anti-ageing prescription.

Clinical evidence supports translational relevance, but with important caveats. The CALERIE trial showed that moderate caloric restriction improves cardiometabolic risk factors and slows the pace of biological ageing measured by composite biomarkers [231,232]. However, this does not establish universal benefit, and biomarker shifts do not necessarily predict long-term gains in function or survival. Excessive restriction may impair bone density, immune competence, wound healing, or physiological reserve [43]. Nutritional interventions, therefore, remain promising, but their optimal use will likely require molecular and clinical stratification.

### 4.3. Exercise, Sleep, and Circadian Regulation as Environmental Modulators

Exercise is one of the most reproducible interventions for improving healthspan and delaying functional decline [43,233,234]. It acts as a systems-level regulator of protein turnover, inflammatory tone, mitochondrial adaptation, and metabolic flexibility. Exercise induces hormetic stress responses that activate cytoprotective pathways, including AMPK, sirtuin-linked programs, autophagy, mitophagy, and antioxidant defense systems [235,236,237]. These effects involve regulators such as *PPARGC1A*/PGC-1α, TFAM, FOXO, and *NFE2L2*/NRF2, which contribute to mitochondrial biogenesis, stress resistance, and proteostatic maintenance. Endurance training promotes autophagy in skeletal muscle and brain, while resistance exercise activates complementary quality-control pathways such as chaperone-assisted selective autophagy [238,239]. These responses are relevant to ageing because they help preserve proteostasis and mitochondrial integrity, both of which decline with age. Exercise also influences inflammatory gene expression, immune cell function, and markers of senescence [43]. Its strongest evidence lies in preserving physiological function, reducing disease burden, and enhancing resilience, rather than proving lifespan extension under all conditions.

Sleep and circadian alignment add another regulatory layer. Circadian rhythms coordinate the temporal organization of metabolism, immune function, autophagy, and hormonal signalling through core clock genes such as *CLOCK*, *ARNTL* (BMAL1), *PER1-3*, *CRY1-CRY2*, and *NR1D1/NR1D2* (REV-ERBα/β), together with downstream oscillatory transcription programs [240,241,242,243]. Sleep disruption and circadian misalignment disturb these programs and are associated with inflammation, metabolic dysfunction, cognitive decline, and accelerated biological ageing [244,245,246]. Experimental evidence further suggests that sleep fragmentation impairs autophagic and proteostatic processes and may contribute to the accumulation of neurodegeneration-associated proteins [247,248,249].

Timing is therefore a regulatory variable in ageing biology. Nutrient exposure and sleep patterns can alter transcriptional timing, while circadian misalignment can disrupt autophagy, inflammation, and metabolic control. Exercise and circadian biology should therefore be framed as gene–environment modulators of ageing-relevant pathways, not merely as generic lifestyle correlates.

### 4.4. Immune, Metabolic, and Microbiome Interfaces

Ageing is accompanied by coordinated changes in immune regulation, energy metabolism, and host–microbe interactions, giving rise to chronic low-grade inflammation, impaired tissue maintenance, and reduced adaptive capacity [250,251]. These domains are interconnected: metabolic states influence inflammatory transcription factors, inflammatory signalling modifies chromatin accessibility, and microbiome-derived metabolites can alter host epigenetic and transcriptional responses. This makes immunometabolism and the microbiome relevant to nutrigenomic models of ageing.

Trained immunity illustrates how environmental exposures can induce durable epigenetic and metabolic reprogramming in innate immune cells [41]. Monocytes and macrophages undergo changes in chromatin accessibility and inflammatory responsiveness, producing long-lasting functional states that resemble memory. In ageing, this is a double-edged phenomenon: some degree of trained immunity may support host defense, but excessive or poorly regulated activation may worsen inflammageing. Because these responses depend on metabolic substrates and epigenetic remodelling, they provide a plausible mechanistic link between nutrition, immune state, and biological ageing.

The gut microbiome extends this interface. Age-associated shifts in microbiome composition have been linked to reduced microbial diversity, altered metabolite production, impaired barrier function, and systemic inflammatory activation [41,42]. Microbiome-derived metabolites such as butyrate can influence host gene expression, immune differentiation, mitochondrial metabolism, and epigenetic regulation [42]. This is an attractive nutrigenomic model, because some biological effects of diet may be mediated not only by direct host nutrient sensing but also by microbial transformation of dietary substrates into signalling molecules.

Translation remains difficult because microbiome studies are strongly affected by cohort heterogeneity, confounding, reverse causation, medication effects, and taxonomic reproducibility problems. It is still often unclear whether ageing-associated microbial signatures are causes, consequences, or correlates of ageing-related decline. For this reason, microbiome-targeted strategies should be framed as investigational components of precision geroscience rather than broadly actionable anti-ageing interventions.

### 4.5. Biological Ageing Clocks, Epigenetic Plasticity, and Molecular Readouts

Epigenetic and proteomic ageing markers have become central to ageing research because they provide molecular estimates of biological age and potential intervention responsiveness [41,252]. They are especially relevant because they capture age-associated changes in DNA methylation, chromatin state, and gene regulation. DNA methylation clocks are built on CpG sites, the methylation state of which changes reproducibly with age and predicts morbidity and mortality [253,254]. More broadly, ageing is associated with epigenetic drift characterized by global hypomethylation and site-specific hypermethylation in regions involved in immune regulation, development, and cellular maintenance [255,256]. Nutritional status, exercise, inflammation, mitochondrial metabolism, and pharmacological interventions may all influence these patterns, which makes epigenetic markers attractive for studying gene–environment interactions over time. Different classes of epigenetic clocks estimate distinct features of ageing biology, including chronological age, mortality risk, or pace of ageing, and should not be treated as interchangeable endpoints.

Proteomic signatures add a complementary layer by capturing downstream physiological consequences of genomic and metabolic regulation [41,257]. Proteins involved in inflammation, metabolic stress, tissue remodelling, and immune signalling often show strong age associations. Emerging markers such as EDA2R may be useful as components of integrated biomarker panels [257]. Mechanistically, EDA2R appears connected to NF-κB- and JNK-related pathways, suggesting involvement in immune and stress-response networks [258,259]. Associations with cognitive decline and metabolic syndrome-related phenotypes further support its possible relevance as a marker of multisystem dysfunction [260]. However, EDA2R should be interpreted as a promising molecular correlate rather than a validated master regulator of ageing.

This subsection now consolidates the biomarker caveat for Section 5: biomarkers are useful for molecular phenotyping, intervention monitoring, and stratification, but favourable clock or proteomic shifts do not automatically demonstrate clinically meaningful slowing of organismal ageing. Most associations between diet, sleep, exercise, and ageing clocks also remain observational, even when biologically coherent [210,211,212,213,214,215,216,217,218,219,220,221,222,223,224,225,226,227,228,229,230,231,232,233,234,235,236,237,238,239,240,241,242,243,244,245,246,247,248,249,250,251,252,253,254,255,256,257,258,259,260,261,262].

### 4.6. Telomere-Centered Translational Interfaces in Ageing Biology

Telomere biology remains relevant to gene–environment modulation because it intersects with nutrient sensing, mitochondrial function, epigenetic regulation, inflammation, and stress adaptation. SIRT1 and SIRT6 act as NAD^+^-sensitive regulators linking metabolic state with chromatin regulation, DNA repair, mitochondrial homeostasis, inflammatory control, and telomere maintenance [263,264,265,266,267,268,269,270,271]. Thus, telomeres are better viewed in this section as integrative response nodes rather than as isolated determinants of ageing.

Telomerase-directed and reprogramming-based approaches remain experimental. TA-65 has been associated with improved immune profiles and reduced inflammatory markers in selected older patient groups [140,287], whereas telomerase gene therapy has shown benefit in experimental models of pulmonary fibrosis and aplastic anaemia [71,72]. Partial epigenetic reprogramming through transient OSKM induction represents a distinct strategy that may reverse selected ageing features, including DNA methylation age and DNA damage, without complete loss of somatic identity [288,289,290,291,292]. These approaches differ substantially in mechanism, maturity, and safety profile and should not be treated as a unified intervention class.

Translational caution is essential because telomere manipulation involves major context-dependent trade-offs. Telomere shortening can promote genomic instability but may also limit malignant proliferation, whereas aberrant telomerase activity can support tumour progression, epithelial–mesenchymal transition, and expansion of mutant clones, including clonal haematopoiesis [293,294,295,296,297,298,299]. Therefore, tissue specificity, cancer risk, baseline telomere status, and dose control are central constraints for any telomere-oriented intervention.

Overall, telomere biology should be presented here as a translational interface and response-stratification node rather than a validated intervention platform. Its relevance lies in linking nutrient sensing, NAD^+^ metabolism, sirtuin activity, inflammation, chromatin regulation, cellular maintenance, and biomarker interpretation [35,36,266]. Current evidence remains too heterogeneous to support broad telomere-directed claims in human ageing interventions.

### 4.7. Behavioural and Psychosocial Modifiers of Ageing-Relevant Regulation

Behavioural and psychosocial factors may also shape ageing trajectories through inflammatory burden, HPA-axis regulation, and long-term biological resilience [46,47,48,49]. This area is less mature than pharmacological or nutrient-sensing interventions, but it remains relevant because chronic stress exposure and environmental support can influence pathways implicated in healthy ageing.

Physical activity is among the most robust modifiers in this domain. Exercise increases *BDNF* expression through DNA methylation changes, histone acetylation, and activation of CaMKII–CREB signalling, enhancing neurogenesis, synaptic plasticity, and cognitive function [163,300]. It also interacts with genetic variation, such as *BDNF* Val66Met, reducing risk of depression and enhancing resilience in susceptible individuals [47]. In addition, exercise influences dopaminergic and glucocorticoid systems, contributing to improved stress regulation and reduced allostatic load [46,300]. These findings link behaviour, neuroplasticity, and biological resilience, although not all evidence is specific to ageing outcomes.

Supportive social environments may also exert measurable biological effects. Positive caregiving, social support, and emotional regulation have been associated with favourable epigenetic profiles in stress-related genes, including *NR3C1* and *OXTR*, which may promote more adaptive HPA-axis regulation [48]. However, social epigenetics remains especially vulnerable to confounding, reverse causation, and inconsistent replication.

Nutrition provides another interface. Dietary methyl donors such as folate, methionine, betaine, and choline influence DNA methylation processes and may affect regulation relevant to brain function and stress response [49]. Nutritional interventions may therefore mitigate some adverse epigenetic effects of chronic stress, particularly during periods of high epigenomic plasticity. Evidence linking these mechanisms directly to healthier ageing trajectories remains less developed than evidence for dietary restriction, exercise, or core metabolic pathways.

Overall, behavioural and psychosocial exposures may modify ageing-relevant biology through stress regulation, inflammatory load, neuroplasticity, and epigenetic responsiveness. Their relevance to healthy ageing is plausible, but causal evidence remains more heterogeneous than for better-established intervention domains.

## 5. Future Directions

Future research should move beyond fragmented approaches that examine longevity-associated variants, epigenetic ageing markers, and anti-ageing interventions in isolation. A more focused agenda should be organized around three unresolved priorities: clarifying biomarker causality, defining predictors of intervention response, and translating nutrigenomic associations into clinically meaningful interventions.

The priority is to distinguish causal ageing biomarkers from correlational indicators of biological or disease-related change. Epigenetic clocks, telomere-related indices, proteomic signatures, and inflammatory or metabolic markers are valuable, but they should not be treated as interchangeable or direct surrogates of slowed organismal ageing [18,23,24,28,252,257,260]. Future studies should determine whether these biomarkers reflect causal ageing mechanisms, adaptive responses, accumulated disease burden, altered cell composition, or tissue-specific vulnerability. This will require multimodal biomarker panels that better capture tissue context, functional relevance, and intervention responsiveness, while also integrating genomics, epigenomics, transcriptomics, metabolomics, microbiome profiling, and longitudinal phenotyping in the same individuals, ideally across multiple tissues and over time [2,3,25,26,28,41,260]. Such approaches are needed to distinguish causal mechanisms from correlational biomarkers and to clarify how inherited susceptibility, environmental exposures, and intervention-responsive pathways interact across the ageing trajectory.

The second priority is to identify the biological and clinical determinants of intervention response. Much of the mechanistic rationale for targeting nutrient sensing, autophagy, mitochondrial quality control, senescence, or chromatin regulation derives from model systems, whereas human findings remain heterogeneous in phenotype definition, tissue specificity, and biomarker interpretation [2,3,28,43]. Future trials should therefore move beyond average treatment effects and incorporate molecular and clinical stratification based on genotype, baseline metabolic state, sex, chronological age, biological age, inflammatory burden, microbiome composition, and epigenetic profile. This will be particularly important for nutrigenomic interventions, since responses to dietary restriction, fasting-related strategies, methyl-donor availability, polyphenols, or microbiome-modulating approaches are unlikely to be uniform across individuals [12,16,29,30,31,32,33,34]. Identifying predictors of response, non-response, and potential harm will be essential for determining which interventions are appropriate for specific biological contexts rather than assuming broad benefit across all individuals [1,2,3,17,23,29,39,40,43].

The third priority is to move nutrigenomics from association-based evidence toward clinically meaningful intervention. Rather than asking whether a single intervention will broadly benefit all individuals, future studies should identify which combinations of genetic background, epigenetic state, metabolic phenotype, and environmental exposure predict meaningful outcomes [1,2,3,17,23,29,39,40,43]. This transition will require longitudinal human trials that integrate tissue-specific or tissue-informed biomarkers, repeated molecular profiling, functional endpoints, and safety monitoring. In this context, the most promising advances are likely to come not from isolated candidate interventions, but from integrated models that link molecular profiling to carefully phenotyped, mechanism-informed, and clinically relevant outcomes. Such a shift would help move the field from biologically plausible associations toward testable, individualized, and safe strategies for promoting healthy ageing and longevity.

## 6. Limitations

This review should be interpreted in light of several methodological considerations. As a narrative review, its aim was to provide a structured and integrative synthesis of current evidence rather than an exhaustive or quantitative assessment. Although the literature search covered major databases and relevant recent publications, study selection and interpretation necessarily involved some degree of author judgement, and the restriction to English-language studies from the last 10 years may have limited the inclusion of some earlier foundational or non-English contributions.

The evidence discussed is also heterogeneous, reflecting the nature of ageing and nutrigenomics research. The review integrates findings from human observational and clinical studies, animal models, and in vitro experiments, which differ in design, biological resolution, and translational relevance. Therefore, mechanistic findings from experimental systems should be interpreted alongside, but not as equivalent to, validated causal evidence in humans. Similarly, biomarkers of biological ageing, including epigenetic clocks, telomere-related measures, inflammatory markers, and proteomic signatures, provide useful molecular information but may capture different aspects of ageing biology and should not be regarded as interchangeable surrogates of organismal ageing.

Finally, because this review emphasizes biological convergence across genetics, epigenetics, nutrition, and ageing-related pathways, it does not provide a formal ranking of interventions, biomarkers, or effect sizes. Many proposed nutrigenomic links remain promising but require further validation through standardized phenotypes, longitudinal multi-omic studies, tissue-informed biomarkers, and better-designed human translational research. Thus, the review is intended as a critical synthesis and conceptual framework to guide future research rather than a definitive evaluation of clinical efficacy.

Taken together, the evidence reviewed here supports an integrated model linking chromatin instability, genome dysfunction, inflammatory signalling, systemic metabolic decline, and modifiable nutrigenomic inputs in the biology of ageing (See Figure 4).

## 7. Conclusions

Human longevity is best understood as a polygenic, environmentally responsive, and biologically stratified trait. Genetic influences are measurable but modest, with *FOXO3* and *APOE* providing the most consistent human signals, while telomere maintenance, nutrient sensing, inflammation, DNA repair, and mitochondrial pathways represent biologically important but more heterogeneous domains.

Epigenetic mechanisms link inherited susceptibility with environmental exposure. DNA methylation, histone modifications, chromatin remodelling, non-coding RNAs, and retrotransposon regulation converge on transcriptional stability, cellular identity, genome maintenance, inflammation, and stress resilience. These mechanisms provide a plausible basis for nutrigenomic modulation, but biomarker changes should not be equated with proven slowing of ageing.

Most ageing-related interventions converge on shared molecular nodes, including AMPK, mTOR, FOXO, SIRT1/SIRT6, NF-κB, NRF2, autophagy, mitochondrial quality control, circadian regulators, and chromatin modifiers. However, their effects are unlikely to be universal and depend on tissue context, age, baseline physiology, disease burden, genetic background, and epigenetic state.

Therefore, precision nutrigeroscience should move beyond universal anti-ageing claims toward stratified, mechanism-based prevention and intervention. Future progress will require human studies that integrate genetics, epigenetics, nutrition, biomarkers, and clinical outcomes to identify who benefits, through which pathways, and under what conditions.

## Data Availability

No new data were created or analyzed in this study.

## References

[1] Bonetti G., Medori M.C., Dhuli K., Macchia A., Donato K., Cristoni S., Miertus S., Miertus J., Veselenyiova D., Iaconelli A. (2023). Nutrigenomics: SNPs correlated to detoxification, antioxidant capacity and longevity. Clin. Ter..

[2] McIntyre R.L., Liu Y.J., Hu M., Morris B.J., Willcox B.J., Donlon T.A., Houtkooper R.H., Janssens G.E. (2022). Pharmaceutical and nutraceutical activation of FOXO3 for healthy longevity. Ageing Res. Rev..

[3] Park J., Peña-Tauber A., Talozzi L., Greicius M.D., Guen Y.L. (2025). Rare genetic associations with human lifespan in UK Biobank are enriched for oncogenic genes. Nat. Commun..

[4] Ji J.S., Liu L., Yan L.L., Zeng Y. (2021). Comparing effects of FOXO3 and residing in urban areas on longevity: A Gene–Environment Interaction study. J. Gerontol. Ser. A.

[5] Marian A.J. (2022). Genetic basis of cardiovascular aging is at the core of human longevity. J. Cardiovasc. Aging.

[6] Panwar V., Singh A., Bhatt M., Tonk R.K., Azizov S., Raza A.S., Sengupta S., Kumar D., Garg M. (2023). Multifaceted role of mTOR (mammalian target of rapamycin) signaling pathway in human health and disease. Signal Transduct. Target. Ther..

[7] Werner H., Laron Z. (2023). Insulin-like growth factors and aging: Lessons from Laron syndrome. Front. Endocrinol..

[8] Lagunas-Rangel F.A. (2022). SIRT7 in the aging process. Cell. Mol. Life Sci..

[9] Korotkov A., Seluanov A., Gorbunova V. (2021). Sirtuin 6: Linking longevity with genome and epigenome stability. Trends Cell Biol..

[10] Kaitsuka T., Matsushita M., Matsushita N. (2021). Regulation of Hypoxic Signaling and Oxidative Stress via the MicroRNA–SIRT2 Axis and Its Relationship with Aging-Related Diseases. Cells.

[11] Fernandes S.A., Demetriades C. (2021). The multifaceted role of nutrient sensing and MTORC1 signaling in physiology and aging. Front. Aging.

[12] Zhu T., Zhao S., Lu N. (2025). Klotho in diabetes mellitus: Research progress and clinical implications. Front. Endocrinol..

[13] Zhang X., Gao Y., Zhang S., Wang Y., Pei X., Chen Y., Zhang J., Zhang Y., Du Y., Hao S. (2025). Mitochondrial dysfunction in the regulation of aging and aging-related diseases. Cell Commun. Signal..

[14] Salminen A. (2022). Role of indoleamine 2,3-dioxygenase 1 (IDO1) and kynurenine pathway in the regulation of the aging process. Ageing Res. Rev..

[15] Huang Y., Che X., Wang P.W., Qu X. (2024). p53/MDM2 signaling pathway in aging, senescence and tumorigenesis. Semin. Cancer Biol..

[16] Gámez-García A., Vazquez B.N. (2021). Nuclear sirtuins and the aging of the immune system. Genes.

[17] Seale K., Horvath S., Teschendorff A., Eynon N., Voisin S. (2022). Making sense of the ageing methylome. Nat. Rev. Genet..

[18] Unnikrishnan A., Freeman W.M., Jackson J., Wren J.D., Porter H., Richardson A. (2018). The role of DNA methylation in epigenetics of aging. Pharmacol. Ther..

[19] Field A.E., Robertson N.A., Wang T., Havas A., Ideker T., Adams P.D. (2018). DNA methylation clocks in aging: Categories, causes, and consequences. Mol. Cell.

[20] Wang K., Liu H., Hu Q., Wang L., Liu J., Zheng Z., Zhang W., Ren J., Zhu F., Liu G.-H. (2022). Epigenetic regulation of aging: Implications for interventions of aging and diseases. Signal Transduct. Target. Ther..

[21] Patel A.B., He Y., Radhakrishnan I. (2023). Histone acetylation and deacetylation–Mechanistic insights from structural biology. Gene.

[22] Yu H., Feng T., Zhang C., Jiao Z., Fan W., Jiang R., Kong D., Li F. (2025). Epigenetic pharmacology in aging: From mechanisms to therapies for age-related disorders. Front. Pharmacol..

[23] Duan R., Fu Q., Sun Y., Li Q. (2022). Epigenetic clock: A promising biomarker and practical tool in aging. Ageing Res. Rev..

[24] Tejedor J.R., Fraga M.F. (2017). Interindividual epigenetic variability: Sound or noise?. BioEssays.

[25] Teschendorff A.E., Horvath S. (2025). Epigenetic ageing clocks: Statistical methods and emerging computational challenges. Nat. Rev. Genet..

[26] Trapp A., Kerepesi C., Gladyshev V.N. (2021). Profiling epigenetic age in single cells. Nat. Aging.

[27] Zhu T., Zheng S.C., Paul D.S., Horvath S., Teschendorff A.E. (2018). Cell and tissue type independent age-associated DNA methylation changes are not rare but common. Aging.

[28] Sillanpää E., Heikkinen A., Kankaanpää A., Paavilainen A., Kujala U.M., Tammelin T.H., Kovanen V., Sipilä S., Pietiläinen K.H., Kaprio J. (2021). Blood and skeletal muscle ageing determined by epigenetic clocks and their associations with physical activity and functioning. Clin. Epigenet..

[29] Wernersson S., Bobby R., Flavell L., Milbradt A.G., Holdgate G.A., Embrey K.J., Akke M. (2022). Bromodomain Interactions with Acetylated Histone 4 Peptides in the BRD4 Tandem Domain: Effects on Domain Dynamics and Internal Flexibility. Biochemistry.

[30] Lozoya O.A., Wang T., Grenet D., Wolfgang T.C., Sobhany M., Da Silva D.G., Riadi G., Chandel N., Woychik R.P., Santos J.H. (2019). Mitochondrial acetyl-CoA reversibly regulates locus-specific histone acetylation and gene expression. Life Sci. Alliance.

[31] Covarrubias A.J., Perrone R., Grozio A., Verdin E. (2021). NAD^+^ metabolism and its roles in cellular processes during ageing. Nat. Rev. Mol. Cell Biol..

[32] Santo E.E., Ribel-Madsen R., Stroeken P.J., De Boer V.C.J., Hansen N.S., Commandeur M., Vaag A.A., Versteeg R., Paik J., Westerhout E.M. (2023). FOXO3A-short is a novel regulator of non-oxidative glucose metabolism associated with human longevity. Aging Cell.

[33] Green C.L., Lamming D.W., Fontana L. (2021). Molecular mechanisms of dietary restriction promoting health and longevity. Nat. Rev. Mol. Cell Biol..

[34] Shabkhizan R., Haiaty S., Moslehian M.S., Bazmani A., Sadeghsoltani F., Bagheri H.S., Rahbarghazi R., Sakhinia E. (2023). The beneficial and adverse effects of autophagic response to caloric restriction and fasting. Adv. Nutr..

[35] Lin J., Epel E. (2021). Stress and telomere shortening: Insights from cellular mechanisms. Ageing Res. Rev..

[36] Wang L., Lu Z., Zhao J., Schank M., Cao D., Dang X., Nguyen L.N., Nguyen L.N.T., Khanal S., Zhang J. (2021). Selective oxidative stress induces dual damage to telomeres and mitochondria in human T cells. Aging Cell.

[37] Pouikli A., Parekh S., Maleszewska M., Nikopoulou C., Baghdadi M., Tripodi I., Folz-Donahue K., Hinze Y., Mesaros A., Hoey D. (2021). Chromatin remodeling due to degradation of citrate carrier impairs osteogenesis of aged mesenchymal stem cells. Nat. Aging.

[38] Jing Y., Jiang X., Ji Q., Wu Z., Wang W., Liu Z., Guillen-Garcia P., Esteban C.R., Reddy P., Horvath S. (2023). Genome-wide CRISPR activation screening in senescent cells reveals SOX5 as a driver and therapeutic target of rejuvenation. Cell Stem Cell.

[39] Alum E., Izah S., Uti D., Ugwu O., Betiang P., Basajja M., Ejemot-Nwadiaro R. (2025). Targeting cellular senescence for healthy aging: Advances in senolytics and senomorphics. Drug Des. Dev. Ther..

[40] Galluzzi L., Pedro J.M.B.-S., Levine B., Green D.R., Kroemer G. (2017). Pharmacological modulation of autophagy: Therapeutic potential and persisting obstacles. Nat. Rev. Drug Discov..

[41] Bulut O., Kilic G., Domínguez-Andrés J. (2021). Immune Memory in Aging: A Wide Perspective Covering Microbiota, Brain, Metabolism, and Epigenetics. Clin. Rev. Allergy Immunol..

[42] Martel J., Ojcius D.M., Ko Y., Chang C., Young J.D. (2019). Antiaging effects of bioactive molecules isolated from plants and fungi. Med. Res. Rev..

[43] Huffman D.M., Schafer M.J., LeBrasseur N.K. (2016). Energetic interventions for healthspan and resiliency with aging. Exp. Gerontol..

[44] Zhu X., Chen Z., Shen W., Huang G., Sedivy J.M., Wang H., Ju Z. (2021). Inflammation, epigenetics, and metabolism converge to cell senescence and ageing: The regulation and intervention. Signal Transduct. Target. Ther..

[45] Sahin E., Colla S., Liesa M., Moslehi J., Müller F.L., Guo M., Cooper M., Kotton D., Fabian A.J., Walkey C. (2011). Telomere dysfunction induces metabolic and mitochondrial compromise. Nature.

[46] Humińska-Lisowska K. (2024). Dopamine in Sports: A narrative review on the genetic and epigenetic factors shaping personality and athletic performance. Int. J. Mol. Sci..

[47] Soler C.T., Kanders S.H., Olofsdotter S., Vadlin S., Åslund C., Nilsson K.W. (2022). Exploration of the Moderating Effects of Physical Activity and Early Life Stress on the Relation between Brain-Derived Neurotrophic Factor (BDNF) rs6265 Variants and Depressive Symptoms among Adolescents. Genes.

[48] Assis S.G., Tavares P.H., Oliveira N., Serpeloni F., Avanci J.Q. (2025). Epigenetics, resilience, Protective Factors and Factors Promoting Positive Outcomes: A scoping review. Int. J. Dev. Neurosci..

[49] Malave L., Van Dijk M.T., Anacker C. (2022). Early life adversity shapes neural circuit function during sensitive postnatal developmental periods. Transl. Psychiatry.

[50] Sebastiani P., Song Z., Ellis D., Tian Q., Schwaiger-Haber M., Stancliffe E., Lustgarten M.S., Funk C.C., Baloni P., Yao C.-H. (2022). A metabolomic signature of the APOE2 allele. GeroScience.

[51] Lozupone M., Dibello V., Sardone R., Castellana F., Zupo R., Lampignano L., Bortone I., Daniele A., Bellomo A., Solfrizzi V. (2023). The impact of apolipoprotein E (APOE) epigenetics on aging and sporadic Alzheimer’s disease. Biology.

[52] Blackburn E.H., Epel E.S., Lin J. (2015). Human telomere biology: A contributory and interactive factor in aging, disease risks, and protection. Science.

[53] Chakravarti D., LaBella K.A., DePinho R.A. (2021). Telomeres: History, health, and hallmarks of aging. Cell.

[54] Blasco M.A. (2005). Telomeres and human disease: Ageing, cancer and beyond. Nat. Rev. Genet..

[55] Armanios M., Blackburn E.H. (2012). The telomere syndromes. Nat. Rev. Genet..

[56] Collins K. (2006). The biogenesis and regulation of telomerase holoenzymes. Nat. Rev. Mol. Cell Biol..

[57] Di Micco R., Krizhanovsky V., Baker D., Di Fagagna F.D. (2020). Cellular senescence in ageing: From mechanisms to therapeutic opportunities. Nat. Rev. Mol. Cell Biol..

[58] López-Otín C., Blasco M.A., Partridge L., Serrano M., Kroemer G. (2023). Hallmarks of aging: An expanding universe. Cell.

[59] Yang J., Luo J., Tian X., Zhao Y., Li Y., Wu X. (2024). Progress in understanding Oxidative Stress, aging, and Aging-Related Diseases. Antioxidants.

[60] Chistiakov D.A., Shkurat T.P., Melnichenko A.A., Grechko A.V., Orekhov A.N. (2017). The role of mitochondrial dysfunction in cardiovascular disease: A brief review. Ann. Med..

[61] Martínez-Zamudio R.I., Dewald H.K., Vasilopoulos T., Gittens-Williams L., Fitzgerald-Bocarsly P., Herbig U. (2021). Senescence-associated β-galactosidase reveals the abundance of senescent CD8+ T cells in aging humans. Aging Cell.

[62] Jiang Y., Li Y., Zhu B. (2015). T-cell exhaustion in the tumor microenvironment. Cell Death Dis..

[63] Martínez P., Blasco M.A. (2011). Telomeric and extra-telomeric roles for telomerase and the telomere-binding proteins. Nat. Rev. Cancer.

[64] Roger L., Tomas F., Gire V. (2021). Mechanisms and regulation of cellular senescence. Int. J. Mol. Sci..

[65] Lim C.J., Cech T.R. (2021). Shaping human telomeres: From shelterin and CST complexes to telomeric chromatin organization. Nat. Rev. Mol. Cell Biol..

[66] Tomás-Loba A., Flores I., Fernández-Marcos P.J., Cayuela M.L., Maraver A., Tejera A., Borrás C., Matheu A., Klatt P., Flores J.M. (2008). Telomerase reverse transcriptase delays aging in Cancer-Resistant mice. Cell.

[67] Jaskelioff M., Muller F.L., Paik J.-H., Thomas E., Jiang S., Adams A.C., Sahin E., Kost-Alimova M., Protopopov A., Cadiñanos J. (2010). Telomerase reactivation reverses tissue degeneration in aged telomerase-deficient mice. Nature.

[68] De Jesus B.B., Vera E., Schneeberger K., Tejera A.M., Ayuso E., Bosch F., Blasco M.A. (2012). Telomerase gene therapy in adult and old mice delays aging and increases longevity without increasing cancer. EMBO Mol. Med..

[69] Muñoz-Lorente M.A., Cano-Martin A.C., Blasco M.A. (2019). Mice with hyper-long telomeres show less metabolic aging and longer lifespans. Nat. Commun..

[70] Shim H.S., Horner J.W., Wu C.-J., Li J., Lan Z.D., Jiang S., Xu X., Hsu W.-H., Zal T., Flores I.I. (2021). Telomerase reverse transcriptase preserves neuron survival and cognition in Alzheimer’s disease models. Nat. Aging.

[71] Povedano J.M., Martinez P., Serrano R., Tejera Á., Gómez-López G., Bobadilla M., Flores J.M., Bosch F., Blasco M.A. (2018). Therapeutic effects of telomerase in mice with pulmonary fibrosis induced by damage to the lungs and short telomeres. eLife.

[72] Bär C., Povedano J.M., Serrano R., Benitez-Buelga C., Popkes M., Formentini I., Bobadilla M., Bosch F., Blasco M.A. (2016). Telomerase gene therapy rescues telomere length, bone marrow aplasia, and survival in mice with aplastic anemia. Blood.

[73] Alder J.K., Armanios M. (2022). Telomere-mediated lung disease. Physiol. Rev..

[74] Farfariello V., Gordienko D.V., Mesilmany L., Touil Y., Germain E., Fliniaux I., Desruelles E., Gkika D., Roudbaraki M., Shapovalov G. (2022). TRPC3 shapes the ER-mitochondria Ca^2+^ transfer characterizing tumour-promoting senescence. Nat. Commun..

[75] Gao X., Yu X., Zhang C., Wang Y., Sun Y., Sun H., Zhang H., Shi Y., He X. (2022). Telomeres and mitochondrial metabolism: Implications for cellular senescence and age-related diseases. Stem Cell Rev. Rep..

[76] Vizioli M.G., Liu T., Miller K.N., Robertson N.A., Gilroy K., Lagnado A.B., Perez-Garcia A., Kiourtis C., Dasgupta N., Lei X. (2020). Mitochondria-to-nucleus retrograde signaling drives formation of cytoplasmic chromatin and inflammation in senescence. Genes Dev..

[77] Li H., Collado M., Villasante A., Strati K., Ortega S., Cañamero M., Blasco M.A., Serrano M. (2009). The Ink4/Arf locus is a barrier for iPS cell reprogramming. Nature.

[78] Marion R.M., Strati K., Li H., Tejera A., Schoeftner S., Ortega S., Serrano M., Blasco M.A. (2009). Telomeres acquire embryonic stem cell characteristics in induced pluripotent stem cells. Cell Stem Cell.

[79] Horvath S. (2013). DNA methylation age of human tissues and cell types. Genome Biol..

[80] Rossiello F., Jurk D., Passos J.F., Di Fagagna F.D. (2022). Telomere dysfunction in ageing and age-related diseases. Nat. Cell Biol..

[81] Titova A., Bilyalov A., Filatov N., Perepechenov S., Kupriyanova D., Brovkin S., Shestakov D., Bodunova N., Gusev O. (2025). Muscle aging heterogeneity: Genetic and structural basis of Sarcopenia resistance. Genes.

[82] Ji J.S., Liu L., Shu C., Yan L.L., Zeng Y. (2021). Sex Difference and Interaction of SIRT1 and FOXO3 candidate longevity genes on life expectancy: A 10-Year Prospective Longitudinal Cohort Study. J. Gerontol. Ser. A.

[83] Hussain S., Yadav S.S., Banerjee M., Usman K., Khattri S. (2021). Evaluation of the Effect of *FOXO3* rs13217795 Genotype and Minor Allele (C) on Clinical Chemistry and Genetic Risk of Diabetes Among the Elderly Individuals from Northern India. Mol. Syndromol..

[84] Chen Q., Aguirre L., Liang G., Zhao H., Dong T., Borrego F., De Rojas I., Hu Q., Reyes C., Su L.-Y. (2024). Identification of a specific APOE transcript and functional elements associated with Alzheimer’s disease. Mol. Neurodegener..

[85] Florczak-Wyspiańska J., Hurła M., Pikor D., Poszwa J., Korczowska-Łącka I., Szymanowicz O., Pluto-Prądzyńska A., Goutor U., Wiszniewska M., Kozubski W. (2025). The APOE gene cluster in normal aging. J. Integr. Neurosci..

[86] Sheikholmolouki E., Sharifi F., Nickhah Z., Vahidi A., Lajevardi R., Haghpanah V., Amoli M.M. (2025). The association of the SIRT6 rs117385980 variant with frailty and longevity: An exploratory study. Sci. Rep..

[87] Zhang J., Zhang Y., You Q., Huang C., Zhang T., Wang M., Zhang T., Yang X., Xiong J., Li Y. (2022). Highly enriched BEND3 prevents the premature activation of bivalent genes during differentiation. Science.

[88] Jain N., Li J.L., Tong L., Jasmine F., Kibriya M.G., Demanelis K., Oliva M., Chen L.S., Pierce B.L. (2024). DNA methylation correlates of chronological age in diverse human tissue types. Epigenet. Chromatin.

[89] Piunti A., Shilatifard A. (2022). Author Correction: The roles of Polycomb repressive complexes in mammalian development and cancer. Nat. Rev. Mol. Cell Biol..

[90] Jadhav U., Manieri E., Nalapareddy K., Madha S., Chakrabarti S., Wucherpfennig K., Barefoot M., Shivdasani R.A. (2020). Replicational Dilution of H3K27me3 in Mammalian Cells and the Role of Poised Promoters. Mol. Cell.

[91] Pérez R.F., Tejedor J.R., Bayón G.F., Fernández A.F., Fraga M.F. (2018). Distinct chromatin signatures of DNA hypomethylation in aging and cancer. Aging Cell.

[92] Yi S.-J., Kim K. (2020). New Insights into the Role of Histone Changes in Aging. Int. J. Mol. Sci..

[93] Yan K., Ji Q., Zhao D., Li M., Sun X., Wang Z., Liu X., Liu Z., Li H., Ding Y. (2023). SGF29 nuclear condensates reinforce cellular aging. Cell Discov..

[94] Sun B., Sherrin M., Roy R. (2022). Unscheduled epigenetic modifications cause genome instability and sterility through aberrant R-loops following starvation. Nucleic Acids Res..

[95] Millán-Zambrano G., Burton A., Bannister A.J., Schneider R. (2022). Histone post-translational modifications—Cause and consequence of genome function. Nat. Rev. Genet..

[96] Zhou S., Liu L., Lu X. (2023). Endogenous retroviruses make aging go viral. Life Med..

[97] Sanz L.A., Hartono S.R., Lim Y.W., Steyaert S., Rajpurkar A., Ginno P.A., Xu X., Chédin F. (2016). Prevalent, Dynamic, and Conserved R-Loop Structures Associate with Specific Epigenomic Signatures in Mammals. Mol. Cell.

[98] Della Valle F., Reddy P., Yamamoto M., Liu P., Saera-Vila A., Bensaddek D., Zhang H., Martinez J.P., Abassi L., Celii M. (2022). LINE-1 RNA causes heterochromatin erosion and is a target for amelioration of senescent phenotypes in progeroid syndromes. Sci. Transl. Med..

[99] Liang C., Ke Q., Liu Z., Ren J., Zhang W., Hu J., Wang Z., Chen H., Xia K., Lai X. (2022). OUP accepted manuscript. Nucleic Acids Res..

[100] Wu Z., Zhang W., Qu J., Liu G.-H. (2024). Emerging epigenetic insights into aging mechanisms and interventions. Trends Pharmacol. Sci..

[101] Turko R., Hajja A., Magableh A.M., Omer M.H., Shafqat A., Khan M.I., Yaqinuddin A. (2025). The emerging role of miRNAs in biological aging and age-related diseases. Non-Coding RNA Res..

[102] Lee R.C., Feinbaum R.L., Ambros V. (1993). The C. elegans heterochronic gene lin-4 encodes small RNAs with antisense complementarity to lin-14. Cell.

[103] Ugalde A.P., Roiz-Valle D., Moledo-Nodar L., Caravia X.M., Freije J.M.P., López-Otín C. (2024). Noncoding RNA contribution to aging and lifespan. J. Gerontol. Ser. A.

[104] Munk R., Panda A.C., Grammatikakis I., Gorospe M., Abdelmohsen K. (2017). Senescence-Associated MicroRNAs. Int. Rev. Cell Mol. Biol..

[105] Mokhberian N., Bolandi Z., Eftekhary M., Hashemi S.M., Jajarmi V., Sharifi K., Ghanbarian H. (2020). Inhibition of miR-34a reduces cellular senescence in human adipose tissue-derived mesenchymal stem cells through the activation of SIRT1. Life Sci..

[106] Ding Z., Ma G., Zhou B., Cheng S., Tang W., Han Y., Chen L., Pang W., Chen Y., Yang D. (2024). Targeting miR-29 mitigates skeletal senescence and bolsters therapeutic potential of mesenchymal stromal cells. Cell Rep. Med..

[107] Hsu C.-C., Chen S.-Y., Ko P.-Y., Kwan F.-C., Su W.-R., Jou I.-M., Wu P.-T. (2024). MicroRNA-146a gene transfer ameliorates senescence and senescence-associated secretory phenotypes in tendinopathic tenocytes. Aging.

[108] Shafqat S., Chicas E.A., Shafqat A., Hashmi S.K. (2022). The Achilles’ heel of cancer survivors: Fundamentals of accelerated cellular senescence. J. Clin. Investig..

[109] Kinser H.E., Pincus Z. (2019). MicroRNAs as modulators of longevity and the aging process. Hum. Genet..

[110] Marttila S., Chatsirisupachai K., Palmer D., De Magalhães J.P. (2019). Ageing-associated changes in the expression of lncRNAs in human tissues reflect a transcriptional modulation in ageing pathways. Mech. Ageing Dev..

[111] Wagner V., Kern F., Hahn O., Schaum N., Ludwig N., Fehlmann T., Engel A., Henn D., Rishik S., Isakova A. (2023). Characterizing expression changes in noncoding RNAs during aging and heterochronic parabiosis across mouse tissues. Nat. Biotechnol..

[112] Catana C.-S., Crișan C.-A., Opre D., Berindan-Neagoe I. (2020). Implications of Long Non-Coding RNAs in Age-Altered proteostasis. Aging Dis..

[113] Ruan L., Mendhe B., Parker E., Kent A., Isales C.M., Hill W.D., McGee-Lawrence M., Fulzele S., Hamrick M.W. (2022). Long non-coding RNA MALAT1 is depleted with Age in Skeletal Muscle in vivo and MALAT1 Silencing Increases Expression of TGF-β1 in vitro. Front. Physiol..

[114] Zhang X., Liu X., Du Z., Wei L., Fang H., Dong Q., Niu J., Li Y., Gao J., Zhang M.Q. (2021). The loss of heterochromatin is associated with multiscale three-dimensional genome reorganization and aberrant transcription during cellular senescence. Genome Res..

[115] Lee J.-H., Kim E.W., Croteau D.L., Bohr V.A. (2020). Heterochromatin: An epigenetic point of view in aging. Exp. Mol. Med..

[116] Yusufova N., Kloetgen A., Teater M., Osunsade A., Camarillo J.M., Chin C.R., Doane A.S., Venters B.J., Portillo-Ledesma S., Conway J. (2020). Histone H1 loss drives lymphoma by disrupting 3D chromatin architecture. Nature.

[117] Paxman J., Zhou Z., O’Laughlin R., Liu Y., Li Y., Tian W., Su H., Jiang Y., Holness S.E., Stasiowski E. (2022). Age-dependent aggregation of ribosomal RNA-binding proteins links deterioration in chromatin stability with challenges to proteostasis. eLife.

[118] Mustafin R.N., Khusnutdinova E.K. (2024). Involvement of transposable elements in Alzheimer’s disease pathogenesis. Vavilov J. Genet. Breed..

[119] Narisu N., Rothwell R., Vrtačnik P., Rodríguez S., Didion J., Zöllner S., Erdos M.R., Collins F.S., Eriksson M. (2019). Analysis of somatic mutations identifies signs of selection during in vitro aging of primary dermal fibroblasts. Aging Cell.

[120] Ostrowski L.A., Hall A.C., Szafranski K.J., Oshidari R., Abraham K.J., Chan J.N.Y., Krustev C., Zhang K., Wang A., Liu Y. (2018). Conserved Pbp1/Ataxin-2 regulates retrotransposon activity and connects polyglutamine expansion-driven protein aggregation to lifespan-controlling rDNA repeats. Commun. Biol..

[121] Gonzales-Ebsen A.C. (2016). Linking telomere loss and mitochondrial dysfunction in chronic disease. Front. Biosci..

[122] Borghini A., Ndreu R., Canale P., Campolo J., Marinaro I., Mercuri A., Turchi S., Andreassi M.G. (2024). Telomere length, mitochondrial DNA, and micronucleus yield in response to oxidative stress in peripheral blood mononuclear cells. Int. J. Mol. Sci..

[123] Menendez J.A., Vellon L., Oliveras-Ferraros C., Cufí S., Vazquez-Martin A. (2011). mTOR-regulated senescence and autophagy during reprogramming of somatic cells to pluripotency: A roadmap from energy metabolism to stem cell renewal and aging. Cell Cycle.

[124] Wang G., Wang B., Yang P. (2022). Epigenetics in congenital heart disease. J. Am. Heart Assoc..

[125] Pal S., Tyler J.K. (2016). Epigenetics and aging. Sci. Adv..

[126] Wang Y., Xu Y., Guo W., Fang Y., Hu L., Wang R., Zhao R., Guo D., Qi B., Ren G. (2022). Ablation of Shank3 alleviates cardiac dysfunction in aging mice by promoting CaMKII activation and Parkin-mediated mitophagy. Redox Biol..

[127] La Torre A., Lo Vecchio F., Greco A. (2023). Epigenetic mechanisms of aging and Aging-Associated diseases. Cells.

[128] Haendeler J., Drose S., Buchner N., Jakob S., Altschmied J., Goy C., Spyridopoulos I., Zeiher A.M., Brandt U., Dimmeler S. (2009). Mitochondrial telomerase reverse transcriptase binds to and protects mitochondrial DNA and function from damage. Arterioscler. Thromb. Vasc. Biol..

[129] Sharma N.K., Reyes A., Green P., Caron M.J., Bonini M.G., Gordon D.M., Holt I.J., Santos J.H. (2011). Human telomerase acts as a hTR-independent reverse transcriptase in mitochondria. Nucleic Acids Res..

[130] Ahmed S., Passos J.F., Birket M.J., Beckmann T., Brings S., Peters H., Birch-Machin M.A., Von Zglinicki T., Saretzki G. (2008). Telomerase does not counteract telomere shortening but protects mitochondrial function under oxidative stress. J. Cell Sci..

[131] Kovalenko O.A., Caron M.J., Ulema P., Medrano C., Thomas A.P., Kimura M., Bonini M.G., Herbig U., Santos J.H. (2010). A mutant telomerase defective in nuclear-cytoplasmic shuttling fails to immortalize cells and is associated with mitochondrial dysfunction. Aging Cell.

[132] Ait-Aissa K., Heisner J.S., Toro L.E.N., Bruemmer D., Doyon G., Harmann L., Geurts A., Camara A.K.S., Beyer A.M. (2019). Telomerase deficiency predisposes to heart failure and Ischemia-Reperfusion injury. Front. Cardiovasc. Med..

[133] Ait-Aissa K., Norwood-Toro L.E., Terwoord J., Young M., Paniagua L.A., Hader S.N., Hughes W.E., Hockenberry J.C., Beare J.E., Linn J. (2022). Noncanonical role of telomerase in regulation of microvascular redox environment with implications for coronary artery disease. Function.

[134] Ale-Agha N., Jakobs P., Goy C., Zurek M., Rosen J., Dyballa-Rukes N., Metzger S., Greulich J., Von Ameln F., Eckermann O. (2021). Mitochondrial telomerase reverse transcriptase protects from myocardial Ischemia/Reperfusion injury by improving complex I composition and function. Circulation.

[135] Chebly A., Khalil C., Kuzyk A., Beylot-Barry M., Chevret E. (2023). T-cell lymphocytes’ aging clock: Telomeres, telomerase and aging. Biogerontology.

[136] Najarro K., Nguyen H., Chen G., Xu M., Alcorta S., Yao X., Zukley L., Metter E.J., Truong T., Lin Y. (2015). Telomere length as an indicator of the robustness of B- and T-Cell response to influenza in older adults. J. Infect. Dis..

[137] Yousefzadeh M.J., Flores R.R., Zhu Y., Schmiechen Z.C., Brooks R.W., Trussoni C.E., Cui Y., Angelini L., Lee K.-A., McGowan S.J. (2021). An aged immune system drives senescence and ageing of solid organs. Nature.

[138] Needham B.L., Wang X., Carroll J.E., Barber S., Sánchez B.N., Seeman T.E., Roux A.V.D. (2018). Sociodemographic correlates of change in leukocyte telomere length during mid- to late-life: The Multi-Ethnic Study of Atherosclerosis. Psychoneuroendocrinology.

[139] Meier H.C.S., Hussein M., Needham B., Barber S., Lin J., Seeman T., Roux A.D. (2019). Cellular response to chronic psychosocial stress: Ten-year longitudinal changes in telomere length in the Multi-Ethnic Study of Atherosclerosis. Psychoneuroendocrinology.

[140] Bawamia B., Spray L., Wangsaputra V.K., Bennaceur K., Vahabi S., Stellos K., Kharatikoopaei E., Ogundimu E., Gale C.P., Keavney B. (2023). Activation of telomerase by TA-65 enhances immunity and reduces inflammation post myocardial infarction. GeroScience.

[141] Fali T., Fabre-Mersseman V., Yamamoto T., Bayard C., Papagno L., Fastenackels S., Zoorab R., Koup R.A., Boddaert J., Sauce D. (2018). Elderly human hematopoietic progenitor cells express cellular senescence markers and are more susceptible to pyroptosis. JCI Insight.

[142] Zanet D.L., Thorne A., Singer J., Maan E.J., Sattha B., Campion A.L., Soudeyns H., Pick N., Murray M., Money D.M. (2014). Association between short leukocyte telomere length and HIV infection in a cohort study: No evidence of a relationship with antiretroviral therapy. Clin. Infect. Dis..

[143] Dowd J.B., Bosch J.A., Steptoe A., Jayabalasingham B., Lin J., Yolken R., Aiello A.E. (2017). Persistent herpesvirus infections and telomere attrition over 3 years in the Whitehall II cohort. J. Infect. Dis..

[144] Liu X., Mo W., Ye J., Li L., Zhang Y., Hsueh E.C., Hoft D.F., Peng G. (2018). Regulatory T cells trigger effector T cell DNA damage and senescence caused by metabolic competition. Nat. Commun..

[145] Plunkett F.J., Franzese O., Finney H.M., Fletcher J.M., Belaramani L.L., Salmon M., Dokal I., Webster D., Lawson A.D.G., Akbar A.N. (2007). The Loss of Telomerase Activity in Highly Differentiated CD8+CD28−CD27− T Cells Is Associated with Decreased Akt (Ser473) Phosphorylation. J. Immunol..

[146] Lanna A., Henson S.M., Escors D., Akbar A.N. (2014). The kinase p38 activated by the metabolic regulator AMPK and scaffold TAB1 drives the senescence of human T cells. Nat. Immunol..

[147] Henson S.M., Lanna A., Riddell N.E., Franzese O., Macaulay R., Griffiths S.J., Puleston D.J., Watson A.S., Simon A.K., Tooze S.A. (2014). p38 signaling inhibits mTORC1-independent autophagy in senescent human CD8+ T cells. J. Clin. Investig..

[148] Lanna A., Coutavas E., Levati L., Seidel J., Rustin M.H.A., Henson S.M., Akbar A.N., Franzese O. (2013). IFN-α Inhibits Telomerase in Human CD8+ T Cells by Both hTERT Downregulation and Induction of p38 MAPK Signaling. J. Immunol..

[149] Campisi J., Di Fagagna F.D. (2007). Cellular senescence: When bad things happen to good cells. Nat. Rev. Mol. Cell Biol..

[150] Wagner K.-D., Wagner N. (2022). The Senescence Markers p16INK4A, p14ARF/p19ARF, and p21 in Organ Development and Homeostasis. Cells.

[151] Betjes M.G.H., Langerak A.W., Van Der Spek A., De Wit E.A., Litjens N.H.R. (2011). Premature aging of circulating T cells in patients with end-stage renal disease. Kidney Int..

[152] Fülöp T., Larbi A., Witkowski J.M. (2019). Human inflammaging. Gerontology.

[153] Ley R.E., Bäckhed F., Turnbaugh P., Lozupone C.A., Knight R.D., Gordon J.I. (2005). Obesity alters gut microbial ecology. Proc. Natl. Acad. Sci. USA.

[154] Zhou Y., Hambly B.D., McLachlan C.S. (2017). FTO associations with obesity and telomere length. J. Biomed. Sci..

[155] De Mello A.H., Costa A.B., Della Giustina Engel J., Rezin G.T. (2017). Mitochondrial dysfunction in obesity. Life Sci..

[156] Wen X., Zhang B., Wu B., Xiao H., Li Z., Li R., Xu X., Li T. (2022). Signaling pathways in obesity: Mechanisms and therapeutic interventions. Signal Transduct. Target. Ther..

[157] Bartelt A., Widenmaier S.B. (2020). Proteostasis in thermogenesis and obesity. Biol. Chem..

[158] Newsholme P., De Bittencourt P.I.H. (2014). The fat cell senescence hypothesis. Curr. Opin. Clin. Nutr. Metab. Care.

[159] Franceschi C. (2017). Obesity in geroscience—Is cellular senescence the culprit?. Nat. Rev. Endocrinol..

[160] Correa-Burrows P., Rogan J., Blanco E., East P., Lozoff B., Gahagan S., Burrows R. (2021). Resolving early obesity leads to a cardiometabolic profile within normal ranges at 23 years old in a two-decade prospective follow-up study. Sci. Rep..

[161] Correa-Burrows P., Burrows R., Albala C., Court F., Salech F., Sanhueza G., Gonzalez-Billault C. (2022). Multiple events case–control study in a prospective cohort to identify systemic, cellular, and molecular biomarkers of obesity-induced accelerated aging in 30-years-olds: The ObAGE study protocol. BMC Geriatr..

[162] Jowf G.I.A., Snijders C., Rutten B.P.F., De Nijs L., Eijssen L.M.T. (2021). The Molecular Biology of Susceptibility to Post-Traumatic Stress Disorder: Highlights of Epigenetics and Epigenomics. Int. J. Mol. Sci..

[163] Schaffner S.L., Kobor M.S. (2022). DNA methylation as a mediator of genetic and environmental influences on Parkinson’s disease susceptibility: Impacts of alpha-Synuclein, physical activity, and pesticide exposure on the epigenome. Front. Genet..

[164] Faraji J., Metz G.A.S. (2026). Environmental epigenetics: New horizons in redefining biological and health outcomes. Environ. Int..

[165] Peña C.J. (2025). Epigenetic regulation of brain development, plasticity, and response to early-life stress. Neuropsychopharmacology.

[166] Banerjee S., S S.M., Banerjee S., Prajwal B.G., Smith C., D’Mello S., Dastidar S.G. (2025). Depression: Epigenetics and epitranscriptomic modifications. World J. Biol. Psychiatry.

[167] Persaud N.S., Cates H.M. (2022). The Epigenetics of Anxiety Pathophysiology: A DNA methylation and Histone Modification focused review. eNeuro.

[168] Scuto M., Rampulla F., Reali G.M., Spanò S.M., Salinaro A.T., Calabrese V. (2024). Hormetic nutrition and redox regulation in Gut–Brain axis disorders. Antioxidants.

[169] Zimmer C., Jimeno B., Martin L.B. (2024). HPA flexibility and FKBP5: Promising physiological targets for conservation. Philos. Trans. R. Soc. B Biol. Sci..

[170] Ochi S., Dwivedi Y. (2022). Dissecting early life stress-induced adolescent depression through epigenomic approach. Mol. Psychiatry.

[171] Bakusic J., Vrieze E., Ghosh M., Bekaert B., Claes S., Godderis L. (2020). Increased methylation of NR3C1 and SLC6A4 is associated with blunted cortisol reactivity to stress in major depression. Neurobiol. Stress.

[172] Peedicayil J. (2023). Genome–Environment Interactions and Psychiatric Disorders. Biomedicines.

[173] Dieckmann L., Czamara D. (2024). Epigenetics of prenatal stress in humans: The current research landscape. Clin. Epigenet..

[174] Vinberg M., McIntyre R., Giraldi A., Coello K. (2024). Struggling can also show on the inside: Current knowledge of the impact of childhood maltreatment on biomarkers in mood disorderss. Neuropsychiatr. Dis. Treat..

[175] Bassil K., Krontira A.C., Leroy T., Escoto A.I.H., Snijders C., Pernia C.D., Pasterkamp R.J., De Nijs L., Van Den Hove D., Kenis G. (2023). In vitro modeling of the neurobiological effects of glucocorticoids: A review. Neurobiol. Stress.

[176] Simonyte S., Grabauskyte I., Macijauskiene J., Lesauskaite V., Lesauskaite V., Kvaal K.S., Stewart R. (2023). Associations of the serotonin transporter gene polymorphism, 5-HTTLPR, and adverse life events with late life depression in the elderly Lithuanian population. Sci. Rep..

[177] Culig L., Chu X., Bohr V.A. (2022). Neurogenesis in aging and age-related neurodegenerative diseases. Ageing Res. Rev..

[178] Bhat A.A., Moglad E., Afzal M., Thapa R., Almalki W.H., Kazmi I., Alzarea S.I., Ali H., Pant K., Singh T.G. (2024). Therapeutic approaches targeting aging and cellular senescence in Huntington’s disease. CNS Neurosci. Ther..

[179] Malekpour M., Shekouh D., Safavinia M.E., Shiralipour S., Jalouli M., Mortezanejad S., Azarpira N., Ebrahimi N.D. (2023). Role of FKBP5 and its genetic mutations in stress-induced psychiatric disorders: An opportunity for drug discovery. Front. Psychiatry.

[180] Cunningham A., Barrett E., Risch S., Lee P.H.U., Lee C., Moghekar A., Patra P., Shim J.W. (2025). NFκB1: A common biomarker linking Alzheimer’s and Parkinson’s disease pathology. Front. Neurosci..

[181] Kirkland J.L., Tchkonia T. (2017). Cellular senescence: A translational perspective. EBioMedicine.

[182] Coperchini F., Greco A., Teliti M., Croce L., Chytiris S., Magri F., Gaetano C., Rotondi M. (2024). Inflamm-ageing: How cytokines and nutrition shape the trajectory of ageing. Cytokine Growth Factor Rev..

[183] Vogler M., Braun Y., Smith V.M., Westhoff M.-A., Pereira R.S., Pieper N.M., Anders M., Callens M., Vervliet T., Abbas M. (2025). The BCL2 family: From apoptosis mechanisms to new advances in targeted therapy. Signal Transduct. Target. Ther..

[184] Qian S., Wei Z., Yang W., Huang J., Yang Y., Wang J. (2022). The role of BCL-2 family proteins in regulating apoptosis and cancer therapy. Front. Oncol..

[185] Jin P., Duan X., Li L., Zhou P., Zou C., Xie K. (2024). Cellular senescence in cancer: Molecular mechanisms and therapeutic targets. MedComm.

[186] Anuar N.N.M., Hisam N.S.N., Liew S.L., Ugusman A. (2020). Clinical review: Navitoclax as a Pro-Apoptotic and Anti-Fibrotic agent. Front. Pharmacol..

[187] Pawge G., Khatik G.L. (2021). p53 regulated senescence mechanism and role of its modulators in age-related disorders. Biochem. Pharmacol..

[188] Mandal R., Kohoutova K., Petrvalska O., Horvath M., Srb P., Veverka V., Obsilova V., Obsil T. (2022). FOXO4 interacts with p53 TAD and CRD and inhibits its binding to DNA. Protein Sci..

[189] Huang Y., He Y., Makarcyzk M.J., Lin H. (2021). Senolytic Peptide FOXO4-DRI Selectively Removes Senescent Cells From in vitro Expanded Human Chondrocytes. Front. Bioeng. Biotechnol..

[190] Hu C., Yang J., Qi Z., Wu H., Wang B., Zou F., Mei H., Liu J., Wang W., Liu Q. (2022). Heat shock proteins: Biological functions, pathological roles, and therapeutic opportunities. MedComm.

[191] Dabravolski S.A., Sukhorukov V.N., Kalmykov V.A., Orekhov N.A., Grechko A.V., Orekhov A.N. (2022). Heat Shock protein 90 as therapeutic target for CVDs and heart ageing. Int. J. Mol. Sci..

[192] Zhang L., Pitcher L.E., Prahalad V., Niedernhofer L.J., Robbins P.D. (2022). Targeting cellular senescence with senotherapeutics: Senolytics and senomorphics. FEBS J..

[193] Miwa S., Kashyap S., Chini E., Von Zglinicki T. (2022). Mitochondrial dysfunction in cell senescence and aging. J. Clin. Investig..

[194] Tavenier J., Nehlin J.O., Houlind M.B., Rasmussen L.J., Tchkonia T., Kirkland J.L., Andersen O., Rasmussen L.J.H. (2024). Fisetin as a senotherapeutic agent: Evidence and perspectives for age-related diseases. Mech. Ageing Dev..

[195] Zhu Y., Doornebal E.J., Pirtskhalava T., Giorgadze N., Wentworth M., Fuhrmann-Stroissnigg H., Niedernhofer L.J., Robbins P.D., Tchkonia T., Kirkland J.L. (2017). New agents that target senescent cells: The flavone, fisetin, and the BCL-XL inhibitors, A1331852 and A1155463. Aging.

[196] Wang Y., Chang J., Liu X., Zhang X., Zhang S., Zhang X., Zhou D., Zheng G. (2016). Discovery of piperlongumine as a potential novel lead for the development of senolytic agents. Aging.

[197] Benameur T., Soleti R., Panaro M.A., La Torre M.E., Monda V., Messina G., Porro C. (2021). Curcumin as Prospective Anti-Aging natural Compound: Focus on brain. Molecules.

[198] Capasso L., De Masi L., Sirignano C., Maresca V., Basile A., Nebbioso A., Rigano D., Bontempo P. (2025). Epigallocatechin gallate (EGCG): Pharmacological properties, biological activities and therapeutic potential. Molecules.

[199] Das D., Banerjee A., Mukherjee S., Maji B.K. (2024). Quercetin inhibits NF-kB and JAK/STAT signaling via modulating TLR in thymocytes and splenocytes during MSG-induced immunotoxicity: An in vitro approach. Mol. Biol. Rep..

[200] Abdelgawad I.Y., Agostinucci K., Sadaf B., Grant M.K.O., Zordoky B.N. (2023). Metformin mitigates SASP secretion and LPS-triggered hyper-inflammation in Doxorubicin-induced senescent endothelial cells. Front. Aging.

[201] Islam M.T., Tuday E., Allen S., Kim J., Trott D.W., Holland W.L., Donato A.J., Lesniewski L.A. (2023). Senolytic drugs, dasatinib and quercetin, attenuate adipose tissue inflammation, and ameliorate metabolic function in old age. Aging Cell.

[202] Sun Y., Qin H., Zhang H., Feng X., Yang L., Hou D.-X., Chen J. (2021). Fisetin inhibits inflammation and induces autophagy by mediating PI3K/AKT/mTOR signaling in LPS-induced RAW264.7 cells. Food Nutr. Res..

[203] Cheng F.-F., Liu Y.-L., Du J., Lin J.-T. (2022). Metformin’s Mechanisms in Attenuating Hallmarks of aging and Age-Related Disease. Aging Dis..

[204] Zhang T., Zhou L., Makarczyk M.J., Feng P., Zhang J. (2025). The Anti-Aging Mechanism of Metformin: From molecular insights to clinical applications. Molecules.

[205] Martin-Montalvo A., Mercken E.M., Mitchell S.J., Palacios H.H., Mote P.L., Scheibye-Knudsen M., Gomes A.P., Ward T.M., Minor R.K., Blouin M.-J. (2013). Metformin improves healthspan and lifespan in mice. Nat. Commun..

[206] Yang L., Lu P., Qi X., Yang Q., Liu L., Dou T., Guan Q., Yu C. (2023). Metformin inhibits inflammatory response and endoplasmic reticulum stress to improve hypothalamic aging in obese mice. iScience.

[207] Kuai Z., Chao X., He Y., Ren W. (2023). Metformin attenuates inflammation and boosts autophagy in the liver and intestine of chronologically aged rats. Exp. Gerontol..

[208] Kodali M., Attaluri S., Madhu L.N., Shuai B., Upadhya R., Gonzalez J.J., Rao X., Shetty A.K. (2021). Metformin treatment in late middle age improves cognitive function with alleviation of microglial activation and enhancement of autophagy in the hippocampus. Aging Cell.

[209] Fahy G.M., Brooke R.T., Watson J.P., Good Z., Vasanawala S.S., Maecker H., Leipold M.D., Lin D.T.S., Kobor M.S., Horvath S. (2019). Reversal of epigenetic aging and immunosenescent trends in humans. Aging Cell.

[210] Quach A., Levine M.E., Tanaka T., Lu A.T., Chen B.H., Ferrucci L., Ritz B., Bandinelli S., Neuhouser M.L., Beasley J.M. (2017). Epigenetic clock analysis of diet, exercise, education, and lifestyle factors. Aging.

[211] Alvers A.L., Wood M.S., Hu D., Kaywell A.C., Dunn W.A., Aris J.P. (2009). Autophagy is required for extension of yeast chronological life span by rapamycin. Autophagy.

[212] Bjedov I., Toivonen J.M., Kerr F., Slack C., Jacobson J., Foley A., Partridge L. (2010). Mechanisms of Life Span Extension by Rapamycin in the Fruit Fly Drosophila melanogaster. Cell Metab..

[213] Schinaman J.M., Rana A., Ja W.W., Clark R.I., Walker D.W. (2019). Rapamycin modulates tissue aging and lifespan independently of the gut microbiota in Drosophila. Sci. Rep..

[214] Hofer S.J., Daskalaki I., Bergmann M., Friščić J., Zimmermann A., Mueller M.I., Abdellatif M., Nicastro R., Masser S., Durand S. (2024). Spermidine is essential for fasting-mediated autophagy and longevity. Nat. Cell Biol..

[215] Eisenberg T., Knauer H., Schauer A., Büttner S., Ruckenstuhl C., Carmona-Gutierrez D., Ring J., Schroeder S., Magnes C., Antonacci L. (2009). Induction of autophagy by spermidine promotes longevity. Nat. Cell Biol..

[216] Xu Y., Xiao W. (2023). NAD^+^: An old but promising therapeutic agent for skeletal muscle ageing. Ageing Res. Rev..

[217] Zhou Q., Zhu L., Qiu W., Liu Y., Yang F., Chen W., Xu R. (2019). Nicotinamide Riboside Enhances Mitochondrial Proteostasis and Adult Neurogenesis through Activation of Mitochondrial Unfolded Protein Response Signaling in the Brain of ALS SOD1G93A Mice. Int. J. Biol. Sci..

[218] Lapierre L.R., Kumsta C., Sandri M., Ballabio A., Hansen M. (2015). Transcriptional and epigenetic regulation of autophagy in aging. Autophagy.

[219] Ryu D., Mouchiroud L., Andreux P.A., Katsyuba E., Moullan N., Nicolet-Dit-Félix A.A., Williams E.G., Jha P., Lo Sasso G., Huzard D. (2016). Urolithin A induces mitophagy and prolongs lifespan in C. elegans and increases muscle function in rodents. Nat. Med..

[220] Singh A., D’Amico D., Andreux P.A., Fouassier A.M., Blanco-Bose W., Evans M., Aebischer P., Auwerx J., Rinsch C. (2022). Urolithin A improves muscle strength, exercise performance, and biomarkers of mitochondrial health in a randomized trial in middle-aged adults. Cell Rep. Med..

[221] Morevati M., Fang E.F., Mace M.L., Kanbay M., Gravesen E., Nordholm A., Egstrand S., Hornum M. (2022). Roles of NAD^+^ in acute and chronic kidney diseases. Int. J. Mol. Sci..

[222] Alves I., Araújo E.M.Q., Dalgaard L.T., Singh S., Børsheim E., Carvalho E. (2025). Protective effects of sulforaphane Preventing inflammation and oxidative stress to Enhance Metabolic health: A Narrative review. Nutrients.

[223] Aaseth J., Alexander J., Alehagen U. (2021). Coenzyme Q10 supplementation–In ageing and disease. Mech. Ageing Dev..

[224] Ruckenstuhl C., Netzberger C., Entfellner I., Carmona-Gutierrez D., Kickenweiz T., Stekovic S., Gleixner C., Schmid C., Klug L., Sorgo A.G. (2014). Lifespan extension by methionine restriction requires Autophagy-Dependent vacuolar acidification. PLoS Genet..

[225] Hansen M., Chandra A., Mitic L.L., Onken B., Driscoll M., Kenyon C. (2008). A Role for Autophagy in the Extension of Lifespan by Dietary Restriction in C. elegans. PLoS Genet..

[226] Gelino S., Chang J.T., Kumsta C., She X., Davis A., Nguyen C., Panowski S., Hansen M. (2016). Intestinal Autophagy Improves Healthspan and Longevity in C. elegans during Dietary Restriction. PLoS Genet..

[227] Yang L., Licastro D., Cava E., Veronese N., Spelta F., Rizza W., Bertozzi B., Villareal D.T., Hotamisligil G.S., Holloszy J.O. (2016). Long-Term calorie restriction enhances cellular Quality-Control processes in human skeletal muscle. Cell Rep..

[228] Jamshed H., Beyl R., Della Manna D., Yang E., Ravussin E., Peterson C. (2019). Early Time-Restricted feeding improves 24-Hour glucose levels and affects markers of the circadian clock, aging, and autophagy in humans. Nutrients.

[229] Gensous N., Garagnani P., Santoro A., Giuliani C., Ostan R., Fabbri C., Milazzo M., Gentilini D., Di Blasio A.M., Pietruszka B. (2020). One-year Mediterranean diet promotes epigenetic rejuvenation with country- and sex-specific effects: A pilot study from the NU-AGE project. GeroScience.

[230] Fiorito G., Caini S., Palli D., Bendinelli B., Saieva C., Ermini I., Valentini V., Assedi M., Rizzolo P., Ambrogetti D. (2021). DNA methylation-based biomarkers of aging were slowed down in a two-year diet and physical activity intervention trial: The DAMA study. Aging Cell.

[231] Ravussin E., Redman L.M., Rochon J., Das S.K., Fontana L., Kraus W.E., Romashkan S., Williamson D.A., Meydani S.N., Villareal D.T. (2015). A 2-Year randomized controlled trial of human caloric Restriction: Feasibility and effects on predictors of health span and longevity. J. Gerontol. Ser. A.

[232] Belsky D.W., Huffman K.M., Pieper C.F., Shalev I., Kraus W.E. (2017). Change in the rate of biological aging in response to caloric restriction: CALERIE Biobank analysis. J. Gerontol. Ser. A.

[233] Halling J.F., Pilegaard H. (2017). Autophagy-Dependent beneficial effects of exercise. Cold Spring Harb. Perspect. Med..

[234] Reimers C.D., Knapp G., Reimers A.K. (2012). Does physical activity increase life expectancy? A review of the literature. J. Aging Res..

[235] Lu X., Chen Y., Shi Y., Shi Y., Su X., Chen P., Wu D., Shi H. (2025). Exercise and exerkines: Mechanisms and roles in anti-aging and disease prevention. Exp. Gerontol..

[236] Kuramoto K., Liang H., Hong J.-H., He C. (2023). Exercise-activated hepatic autophagy via the FN1-α5β1 integrin pathway drives metabolic benefits of exercise. Cell Metab..

[237] Chen Y., Ma Y., Tang J., Zhang D., Zhao Q., Liu J., Tang H., Zhang J., He G., Zhong C. (2023). Physical exercise attenuates age-related muscle atrophy and exhibits anti-ageing effects via the adiponectin receptor 1 signalling. J. Cachexia Sarcopenia Muscle.

[238] Tedesco B., Vendredy L., Timmerman V., Poletti A. (2023). The chaperone-assisted selective autophagy complex dynamics and dysfunctions. Autophagy.

[239] Ulbricht A., Gehlert S., Leciejewski B., Schiffer T., Bloch W., Höhfeld J. (2015). Induction and adaptation of chaperone-assisted selective autophagy CASA in response to resistance exercise in human skeletal muscle. Autophagy.

[240] Duffy J.F., Zitting K.-M., Chinoy E.D. (2015). Aging and circadian rhythms. Sleep Med. Clin..

[241] Stenvers D.J., Scheer F.a.J.L., Schrauwen P., La Fleur S.E., Kalsbeek A. (2018). Circadian clocks and insulin resistance. Nat. Rev. Endocrinol..

[242] Cao R. (2018). MTOR signaling, translational control, and the circadian clock. Front. Genet..

[243] Jordan S.D., Lamia K.A. (2012). AMPK at the crossroads of circadian clocks and metabolism. Mol. Cell. Endocrinol..

[244] Ebata H., Hansen M. (2026). Links between autophagy and healthy aging. J. Mol. Biol..

[245] Guo X., Keenan B.T., Sarantopoulou D., Lim D.C., Lian J., Grant G.R., Pack A.I. (2019). Age attenuates the transcriptional changes that occur with sleep in the medial prefrontal cortex. Aging Cell.

[246] McKillop L.E., Vyazovskiy V.V. (2020). Sleep and ageing: From human studies to rodent models. Curr. Opin. Physiol..

[247] Rostamzadeh F., Joukar S., Yeganeh-Hajahmadi M. (2024). The role of Klotho and sirtuins in sleep-related cardiovascular diseases: A review study. npj Aging.

[248] Blum I.D., Keleş M.F., Baz E.-S., Han E., Park K., Luu S., Issa H., Brown M., Ho M.C.W., Tabuchi M. (2020). Astroglial calcium signaling encodes sleep need in drosophila. Curr. Biol..

[249] Xie Y., Ba L., Wang M., Deng S., Chen S., Huang L., Zhang M., Wang W., Ding F. (2019). Chronic sleep fragmentation shares similar pathogenesis with neurodegenerative diseases: Endosome-autophagosome-lysosome pathway dysfunction and microglia-mediated neuroinflammation. CNS Neurosci. Ther..

[250] Pereira B., Xu X.-N., Akbar A.N. (2020). Targeting inflammation and immunosenescence to improve vaccine responses in the elderly. Front. Immunol..

[251] Rubio-Ruiz M.E., Peredo-Escárcega A.E., Cano-Martínez A., Guarner-Lans V. (2015). An evolutionary perspective of nutrition and inflammation as mechanisms of cardiovascular disease. Int. J. Evol. Biol..

[252] Johnson A.A., English B.W., Shokhirev M.N., Sinclair D.A., Cuellar T.L. (2022). Human age reversal: Fact or fiction?. Aging Cell.

[253] Valenzuela H.F., Fuller T., Edwards J., Finger D., Molgora B. (2009). Cycloastragenol extends T cell proliferation by increasing telomerase activity (90.30). J. Immunol..

[254] Valenzuela H., Burguez C., Chikami K., Cruz M., Rinaldi D. (2013). Assessing natural telomerase activators (P4342). J. Immunol..

[255] Molgora B., Bateman R., Sweeney G., Finger D., Dimler T., Effros R., Valenzuela H. (2013). Functional assessment of pharmacological telomerase activators in human T cells. Cells.

[256] Fauce S.R., Jamieson B.D., Chin A.C., Mitsuyasu R.T., Parish S.T., Ng H.L., Kitchen C.M.R., Yang O.O., Harley C.B., Effros R.B. (2008). Telomerase-Based pharmacologic enhancement of antiviral function of human CD8+ T lymphocytes. J. Immunol..

[257] Farrington G., Tonge L., Branagan T., Sudirman S., Fang C., Luk L., Kir S., Bolis M., Ahmetov I.I., Ross K. (2025). The roles of EDA2R in Ageing and Disease. Aging Cell.

[258] Sinha S.K., Zachariah S., Quiñones H.I., Shindo M., Chaudhary P.M. (2002). Role of TRAF3 and -6 in the Activation of the NF-κB and JNK Pathways by X-linked Ectodermal Dysplasia Receptor. J. Biol. Chem..

[259] Yang R., Mei Y., Jiang Y., Li H., Zhao R., Sima J., Yao Y. (2022). Ectodysplasin A (EDA) signaling: From skin appendage to multiple diseases. Int. J. Mol. Sci..

[260] Margara-Escudero H.J., Paz-Graniel I., García-Gavilán J.F., Fitó M., Bralten J., Matura S., Glennon J.C., Schiweck C., Vilella E., Santos-Lozano J.M. (2025). Plasma neurology-related proteins associated with cognition are modulated by lifestyle in adults. EBioMedicine.

[261] Lehallier B., Shokhirev M.N., Wyss-Coray T., Johnson A.A. (2020). Data mining of human plasma proteins generates a multitude of highly predictive aging clocks that reflect different aspects of aging. Aging Cell.

[262] Gao X., Huang N., Guo X., Huang T. (2022). Role of sleep quality in the acceleration of biological aging and its potential for preventive interaction on air pollution insults: Findings from the UK Biobank cohort. Aging Cell.

[263] Haigis M.C., Sinclair D.A. (2010). Mammalian sirtuins: Biological Insights and disease Relevance. Annu. Rev. Pathol. Mech. Dis..

[264] Houtkooper R.H., Pirinen E., Auwerx J. (2012). Sirtuins as regulators of metabolism and healthspan. Nat. Rev. Mol. Cell Biol..

[265] Bellet M.M., Nakahata Y., Boudjelal M., Watts E., Mossakowska D.E., Edwards K.A., Cervantes M., Astarita G., Loh C., Ellis J.L. (2013). Pharmacological modulation of circadian rhythms by synthetic activators of the deacetylase SIRT1. Proc. Natl. Acad. Sci. USA.

[266] Da Cunha M., Arruda S. (2017). Tucum-do-Cerrado (Bactris setosa Mart.) May Promote Anti-Aging Effect by Upregulating SIRT1-Nrf2 Pathway and Attenuating Oxidative Stress and Inflammation. Nutrients.

[267] Lee S.-H., Lee J.-H., Lee H.-Y., Min K.-J. (2019). Sirtuin signaling in cellular senescence and aging. BMB Rep..

[268] Lee S.E., Lee S.B., Roh J.-I., Kim K.P., Lee J.H., Lee H.-W. (2024). SIRT1 regulates the localization and stability of telomerase protein by direct interaction. Biochem. Biophys. Res. Commun..

[269] Cui H., Yang W., He S., Chai Z., Wang L., Zhang G., Zou P., Sun L., Yang H., Chen Q. (2023). TERT transcription and translocation into mitochondria regulate benzo[a]pyrene/BPDE-induced senescence and mitochondrial damage in mouse spermatocytes. Toxicol. Appl. Pharmacol..

[270] Amano H., Chaudhury A., Rodriguez-Aguayo C., Lu L., Akhanov V., Catic A., Popov Y.V., Verdin E., Johnson H., Stossi F. (2019). Telomere Dysfunction Induces Sirtuin Repression that Drives Telomere-Dependent Disease. Cell Metab..

[271] Mohammed S., Thadathil N., Selvarani R., Nicklas E.H., Wang D., Miller B.F., Richardson A., Deepa S.S. (2021). Necroptosis contributes to chronic inflammation and fibrosis in aging liver. Aging Cell.

[272] Kawahara T.L.A., Michishita E., Adler A.S., Damian M., Berber E., Lin M., McCord R.A., Ongaigui K.C.L., Boxer L.D., Chang H.Y. (2009). SIRT6 links Histone H3 lysine 9 deacetylation to NF-ΚB-Dependent Gene Expression and organismal life span. Cell.

[273] Howitz K.T., Bitterman K.J., Cohen H.Y., Lamming D.W., Lavu S., Wood J.G., Zipkin R.E., Chung P., Kisielewski A., Zhang L.-L. (2003). Small molecule activators of sirtuins extend Saccharomyces cerevisiae lifespan. Nature.

[274] Bonkowski M.S., Sinclair D.A. (2016). Slowing ageing by design: The rise of NAD^+^ and sirtuin-activating compounds. Nat. Rev. Mol. Cell Biol..

[275] Dai H., Sinclair D.A., Ellis J.L., Steegborn C. (2018). Sirtuin activators and inhibitors: Promises, achievements, and challenges. Pharmacol. Ther..

[276] Wang Y., He J., Liao M., Hu M., Li W., Ouyang H., Wang X., Ye T., Zhang Y., Ouyang L. (2018). An overview of Sirtuins as potential therapeutic target: Structure, function and modulators. Eur. J. Med. Chem..

[277] Iside C., Scafuro M., Nebbioso A., Altucci L. (2020). SIRT1 activation by Natural Phytochemicals: An Overview. Front. Pharmacol..

[278] Nikseresht S., Khodagholi F., Ahmadiani A. (2018). Protective effects of ex-527 on cerebral ischemia–reperfusion injury through necroptosis signaling pathway attenuation. J. Cell. Physiol..

[279] Huang J., Tian R., Yang Y., Jiang R., Dai J., Tang L., Zhang L. (2017). The SIRT1 inhibitor EX-527 suppresses mTOR activation and alleviates acute lung injury in mice with endotoxiemia. Innate Immun..

[280] Smith M.R., Syed A., Lukacsovich T., Purcell J., Barbaro B.A., Worthge S.A., Wei S.R., Pollio G., Magnoni L., Scali C. (2014). A potent and selective Sirtuin 1 inhibitor alleviates pathology in multiple animal and cell models of Huntington’s disease. Hum. Mol. Genet..

[281] Brandl L., Kirstein N., Neumann J., Sendelhofert A., Vieth M., Kirchner T., Menssen A. (2018). The c-MYC/NAMPT/SIRT1 feedback loop is activated in early classical and serrated route colorectal cancer and represents a therapeutic target. Med. Oncol..

[282] Gomes A.P., Price N.L., Ling A.J.Y., Moslehi J.J., Montgomery M.K., Rajman L., White J.P., Teodoro J.S., Wrann C.D., Hubbard B.P. (2013). Declining NAD^+^ Induces a Pseudohypoxic State Disrupting Nuclear-Mitochondrial Communication during Aging. Cell.

[283] Stock A.J., Liu Y. (2021). NAD-Linked Metabolism and Intervention in short telomere syndromes and murine models of telomere dysfunction. Front. Aging.

[284] Diman A., Boros J., Poulain F., Rodriguez J., Purnelle M., Episkopou H., Bertrand L., Francaux M., Deldicque L., Decottignies A. (2016). Nuclear respiratory factor 1 and endurance exercise promote human telomere transcription. Sci. Adv..

[285] Liu J., Ge Y., Wu S., Ma D., Xu W., Zhang Y., Yang Y. (2019). Association between antidiabetic agents use and leukocyte telomere shortening rates in patients with type 2 diabetes. Aging.

[286] Hachmo Y., Hadanny A., Hamed R.A., Daniel-Kotovsky M., Catalogna M., Fishlev G., Lang E., Polak N., Doenyas K., Friedman M. (2020). Hyperbaric oxygen therapy increases telomere length and decreases immunosenescence in isolated blood cells: A prospective trial. Aging.

[287] Su X., Wang C., Gou Z., Qu Y. (2025). Effects of TA-65 on telomere length, functional outcomes, and inflammation: A systematic review and meta-analysis. Cell Biol. Toxicol..

[288] Ocampo A., Reddy P., Martinez-Redondo P., Platero-Luengo A., Hatanaka F., Hishida T., Li M., Lam D., Kurita M., Beyret E. (2016). In vivo amelioration of Age-Associated hallmarks by partial reprogramming. Cell.

[289] Browder K.C., Reddy P., Yamamoto M., Haghani A., Guillen I.G., Sahu S., Wang C., Luque Y., Prieto J., Shi L. (2022). In vivo partial reprogramming alters age-associated molecular changes during physiological aging in mice. Nat. Aging.

[290] Lu Y., Brommer B., Tian X., Krishnan A., Meer M., Wang C., Vera D.L., Zeng Q., Yu D., Bonkowski M.S. (2020). Reprogramming to recover youthful epigenetic information and restore vision. Nature.

[291] Gill D., Parry A., Santos F., Okkenhaug H., Todd C.D., Hernando-Herraez I., Stubbs T.M., Milagre I., Reik W. (2022). Multi-omic rejuvenation of human cells by maturation phase transient reprogramming. eLife.

[292] Chondronasiou D., Gill D., Mosteiro L., Urdinguio R.G., Berenguer-Llergo A., Aguilera M., Durand S., Aprahamian F., Nirmalathasan N., Abad M. (2022). Multi-omic rejuvenation of naturally aged tissues by a single cycle of transient reprogramming. Aging Cell.

[293] López-Otín C., Pietrocola F., Roiz-Valle D., Galluzzi L., Kroemer G. (2023). Meta-hallmarks of aging and cancer. Cell Metab..

[294] Bilgili H., Białas A.J., Górski P., Piotrowski W.J. (2019). Telomere abnormalities in the pathobiology of idiopathic pulmonary fibrosis. J. Clin. Med..

[295] Merkt W., Bueno M., Mora A.L., Lagares D. (2019). Senotherapeutics: Targeting senescence in idiopathic pulmonary fibrosis. Semin. Cell Dev. Biol..

[296] Molina-Molina M., Borie R. (2018). Clinical implications of telomere dysfunction in lung fibrosis. Curr. Opin. Pulm. Med..

[297] Selman M., Buendía-Roldán I., Pardo A. (2016). Aging and pulmonary fibrosis. Rev. Investig. Clínica.

[298] Lee-Chang C., Bodogai M., Moritoh K., Chen X., Wersto R., Sen R., Young H.A., Croft M., Ferrucci L., Biragyn A. (2016). Aging Converts Innate B1a Cells into Potent CD8+ T Cell Inducers. J. Immunol..

[299] Kim E., Rebecca V., Fedorenko I.V., Messina J.L., Mathew R., Maria-Engler S.S., Basanta D., Smalley K.S.M., Anderson A.R.A. (2013). Senescent Fibroblasts in Melanoma Initiation and progression: An Integrated theoretical, experimental, and Clinical approach. Cancer Res..

[300] Mănescu D.C. (2026). Training load oscillation and epigenetic plasticity: Molecular pathways connecting energy metabolism and athletic personality. Int. J. Mol. Sci..

